# The influence of the inactives subset generation on the performance of machine learning methods

**DOI:** 10.1186/1758-2946-5-17

**Published:** 2013-04-05

**Authors:** Sabina Smusz, Rafał Kurczab, Andrzej J Bojarski

**Affiliations:** 1 Department of Medicinal Chemistry, Institute of Pharmacology, Polish Academy of Sciences, Smętna 12, Kraków, 31-343, Poland; 2 Faculty of Chemistry, Jagiellonian University, R. Ingardena 3, Kraków, 30-060, Poland

## Abstract

**Background:**

A growing popularity of machine learning methods application in virtual screening, in both classification and regression tasks, can be observed in the past few years. However, their effectiveness is strongly dependent on many different factors.

**Results:**

In this study, the influence of the way of forming the set of inactives on the classification process was examined: random and diverse selection from the ZINC database, MDDR database and libraries generated according to the DUD methodology. All learning methods were tested in two modes: using one test set, the same for each method of inactive molecules generation and using test sets with inactives prepared in an analogous way as for training. The experiments were carried out for 5 different protein targets, 3 fingerprints for molecules representation and 7 classification algorithms with varying parameters. It appeared that the process of inactive set formation had a substantial impact on the machine learning methods performance.

**Conclusions:**

The level of chemical space limitation determined the ability of tested classifiers to select potentially active molecules in virtual screening tasks, as for example DUDs (widely applied in docking experiments) did not provide proper selection of active molecules from databases with diverse structures. The study clearly showed that inactive compounds forming training set should be representative to the highest possible extent for libraries that undergo screening.

## Background

Machine learning methods are among the most popular tools used in cheminformatic tasks [1-3]. So far, many aspects of their application in experiments connected with the classification of chemical compounds have been extensively examined: the type of molecules representation [4], number of compounds from particular class in the dataset [5], parameters of learning algorithms [6], the type of machine learning method [7], etc. Interestingly, the influence of differences in dataset composition resulting from various ways of selection of molecules forming a set of inactives has never been thoroughly investigated.

Databases of compounds with reported activity towards particular target usually contain only a few molecules which are proved to be inactive. Therefore, during the preparation for machine learning experiments, the need of generating sets of compounds assumed as inactive arises. Various approaches to this task have already been proposed. Selection from databases of known ligands [8,9], where compounds with unconfirmed activity towards considered receptor (active towards proteins other than the target of the interest) were assumed as inactive, generation of putative inactives [10], random selection out of large databases [7] are just some the most common examples. Only in very few cases, number of inactive compounds is sufficient enough to perform ML experiments [11].

In this study, six most frequently used ways of selecting assumed inactives were tested: random and diverse selection from: the ZINC database [12], the MDDR database [13] and libraries generated according to the DUD methodology [14] in terms of their impact on the machine learning methods performance.

As the common sense suggest, such effect should be observed, but to determine if it is noticeable and repeatable (and thus dependent on the experimental conditions) all tests were performed for 5 different protein targets, with the use of 3 different fingerprints for molecules representation and 7 machine learning algorithms with varying parameters.

## Results

All experiments were performed for wide spectrum of parameters of machine learning methods. The presented results are related only to those settings that provided the highest classification efficiency in the most of cases (bolded in Table 1); an exemplary panel of graphs with full results for every machine learning algorithm is available in Additional file 1: Figure S1.

**Table 1:** Machine learning methods used in the experiments with the optional abbreviations used in further work

**Classifier**	**Classification scheme**	**Parameters**
Naïve Bayes (NB)	bayes	-
Sequential Minimal Optimization (SMO)	functions	The complexity parameter was set at 1, the epsilon for a round-off error was 1.0 E-12, and an option of normalizing training data was chosen.
		Kernels:
		** 1) The normalized polynomial kernel**,
		2) The polynomial kernel
		3) The RBF kernel
Instance-Based Learning (Ibk)	lazy	The brute force search algorithm for nearest neighbour search with Euclidean distance function.
		The number of neighbours used:
		1) **1**
		2) 5
		3) 10
		4) 20
Decorate	meta	One artificial example used during training, number of member classifiers in the Decorate ensemble: 10, the maximum number of iterations: 10.
		Base classifiers:
		1) **NaïveBayes**
		2) J48
Hyperpipes	misc	-
J48	trees	1) With reduced-error pruning
		2) **With C.4.5 pruning**
Random Forest (RF)	trees	Trees with unlimited depth, seed number: 1.
		Number of generated trees:
		1) 5
		2) 10
		3) 50
		4) **100**

Bolded parameters correspond with the one providing the best results for particular machine learning method (see Results section & Additional file 1: Figure S1).

Similarly to already reported findings, the number of trees grown when Random Forest is run equal to 100 led to the highest classification efficiency [6], as well as setting the number of neighbours to 1 when k-NN is applied [5]. Some authors also pointed out, that RBF kernel is an optimal choice for SVM experiments [6], whereas in our tests, the normalized polykernel showed the best values of evaluating parameters for the majority of cases. Naïve Bayes appeared to be more effective base classifier than J48, and C.4.5 pruning won over the reduced-error pruning (Table 1, Additional File 1: Figure S1).

**Figure 1:**
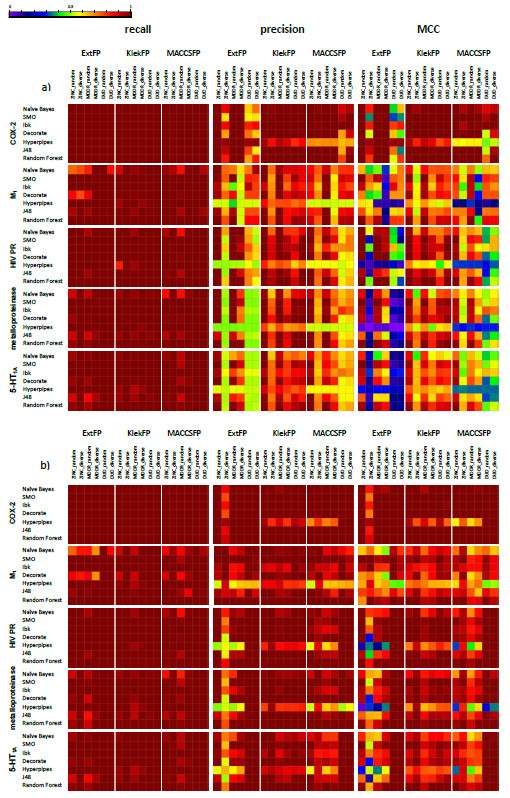
**A set of heat maps visualizing the values of evaluating parameters obtained in a) common-test set mode experiments and in b) various-test set mode experiments.** Figure 1 presents recall, precision and MCC values obtained in the experiments. Columns of maps are referring to particular evaluating parameter, rows to particular target. Rows in maps correspond with different machine learning methods, whereas columns in maps refer to different training sets and use of various fingerprints for molecules representation.

Due to a great number of results, the heat maps (Figure 1) were employed to qualitatively represent values of the evaluating parameters (all numerical values together with their standard deviations are available in Additional file 2: Tables S1–S3). In case where subsets of inactives were selected randomly, the presented results are the averaged outcomes from 10 iterations (standard deviation of evaluating parameters values did not exceeded 0.05, confirming the consistency of the shown data).

### Common-test set mode

The influence of the way of inactive molecules selection on machine learning methods performance was similar for different algorithms (Figure 1a). All classifiers were able to correctly indicate active molecules, regardless of the changes in the inactives set composition, which is shown by the dark-red maps referring to this evaluating parameter. On the other hand, precision was very sensitive to variations in the way of subsets of inactives generation – heat maps related to this parameter are definitely more complex comparing to those presenting recall values. Changes in precision values also had a major contribution to variations in MCC – they were even more visible due to the range of MCC from −1 to 1. For this reason, both of these parameters are discussed together.

For all classifiers, the highest effectiveness of classification occurred for ZINC_random sets (first columns in each part of heat maps referring to particular fingerprint). Although in this case, the test set was generated in the analogous way as for training, which makes it a little bit privileged, this type of test set is a very good illustration of the virtual screening experiment, where large libraries of chemical structures are evaluated. Therefore, this part of study can be regarded as a reference point for the rest of results. For the experiments with COX-2 inhibitors, the MCC and precision values were similar to those obtained for ZINC_random also for both sets of inactives selected out of the MDDR database when MACCSFP and ExtFP were used. Applying KlekFP led to situation where those parameters remained on similar level for all classifiers but Hyperpipes (lower by ~0.3 for precision and ~0.5 for MCC comparing to ZINC_random). For the rest of targets, changes in precision and MCC (being a consequence of varying conditions of inactives selection) were very similar. Their highest values (very close to ZINC_random) were provided by random selection of compounds from the MDDR database and the lowest by DUD sets, which caused the reduction of classification effectiveness even by ~0.5 or ~0.8 for precision and MCC respectively). In addition, similarly to COX-2, the lowest precision and MCC changes for different sets of inactives occurred when molecules were represented by KlekFP. All of the performed experiments indicated that random selection of inactives provided better results than the diverse approach. This dependence was the most clearly indicated in case of “ZINC sets” of inactives - up to ~0.8 variation in MCC values, for inactives from the MDDR database this difference was around 0.2, whereas for “DUD sets” those two methods of selecting inactive molecules led to comparable results, although they were significantly less efficient than those obtained with the “inactives” selected from the two previous databases.

### Various-test sets mode

As regards to the results obtained in the various-test sets mode (Figure 1b), differences between particular experiments were suppressed. It is hard to identify the set providing the best recall or the one that led to significant fall in its values. Even though, for some methods (such as NB, Decorate and J48) a slight drop was observed for ZINC_random and MDDR_random sets indicated by shifting in colours of boxes corresponding to experiments with its use from dark to light red.

In this part of research, the rate of false positives was significantly lower, which is illustrated by the dark red precision maps in Figure 1b) versus the light red-yellow ones corresponding to experiments with the common-test set mode (Figure 1a), and this also had a consequence in higher MCC values. An apparent fall in values of these parameters occurred for experiments with datasets containing inactives picked diversely from the ZINC database and represented by ExtFP, as well as for application of Hyperpipes as a classifier. The highest evaluating parameters values were obtained for DUD sets as it is shown by dark-red colour of two last columns in heat maps generated for each fingerprint. In case of using KlekFP, ZINC_diverse sets also led to high values of evaluating parameters, regardless of the machine learning algorithm applied for the classification task.

### PubChem experiments

As inactives selected from PubChem database much more resembles the second part of the study (various-test sets mode) – the chemical space of inactive molecules is much more limited comparing to ZINC database or any other commercially available library of compounds, results obtained for this external validation sets coincide with those obtained in the various-test set mode. Heat maps and numerical values of evaluating parameters corresponding to them are available in Additional file 3: Figure S2, Additional file 4: Table S4.

### Influence of the inactives subset composition on particular machine learning method performance

**Figure 2:**
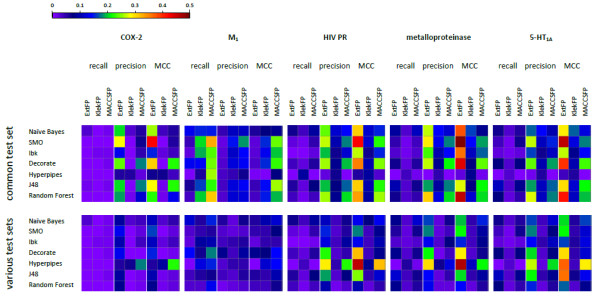
**A set of heat maps visualizing the values of standard deviation of evaluating parameters for experiments with the use of sets with variously generated inactive compounds.** Figure 2 presents standard deviation of recall, precision and MCC values obtained in the experiments. Columns of maps refer to particular target, rows to common-test and various-test sets mode respectively. Rows in maps correspond with different machine learning methods, whereas columns in maps refer to different parameters and fingerprints.

Although, in general the results are comparable and consistent between learning algorithms, there are such ones that are more sensitive to the inactive set composition. To simplify the results interpretation, the influence of inactives selection on machine learning methods was expressed by standard deviation (SD) of evaluating parameters values for experiments with inactive subsets generated in different ways (Figure 2).

In general, changes in recall, precision and MCC were the most significant when the machine learning methods were combined with ExtFP (standard deviation of MCC was even close to 0.4 as it is shown by red spots on heat maps). On the other hand, the most stable results were provided by KlekFP – SD of MCC values was more than 0.2 lower compared to already mentioned tests.

Except the classification of M_1_ agonists, recall values were not much influenced by changes in dataset composition. It remained on the similar level for all experiments with SD usually below 0.1, which is expressed by corresponding dark blue squares on heat maps (Figure 2). Changes in precision values contributed the most to MCC fluctuations and the tendencies in variations of these two parameters were very similar. Even in case of experiments with COX-2 inhibitors, when all values of evaluating parameters were on high level, the MCC changes (resulting from different ways of set of inactives composition) were clearly visible and reached the maximum value of 0.36 for SMO.

SMO algorithm was the most dependent on the way of the inactives set construction (indicated by red spots on the heat maps corresponding to SD of MCC values from ~0.3–0.4). Two methods least affected by inactives set formation were Hyperpipes (SD < 0.1), and Ibk (SD equal 0.2 at most), yet the first one was at the same time the worst classifier in terms of MCC (MCC of 0.1 using ExtFP for molecules representation).

## Discussion

As it appears from the obtained results the size and properties of the chemical space, that is used for the experiments has undoubtedly a great impact on the performance of machine learning methods. All tested classifiers faced the problem of correct classification of compounds picked up from the ZINC database when they were trained on sets with inactives selected according to different approaches (from MDDR database or DUD libraries). Although the ability of machine learning methods to select active molecules was on a very similar level (recall values were not varying a lot when moving to different sets), the high rate of false positives indicates errors in assigning a proper class label to inactive compounds. Strong limitation of chemical space (as it happen in DUD libraries) led to variation of precision values at the level of 0.5–0.7. The larger the database from which the compounds were selected, the more significant the improvement of the results. What is also worth noting, the evaluating parameters values were much higher when molecules for inactives set formation were picked up randomly, rather than with the assurance of the maximum diversity of the selected compounds. The values of MCC indicate that definitely the best results were obtained for sets with inactives randomly selected out of the ZINC database.

The case is different when inactives for test sets formation were selected in an analogous way as compounds chosen for training. In those experiments, the dependencies are reverse comparing to the common-test set mode. In this case, the best results were provided by sets with compounds selected from libraries containing smaller number of molecules (the MCC values exceeding even 0.9 for “DUD sets”). In contrast to the outcomes from the common-test set mode, better results were also obtained when compounds were selected in a diverse way rather than random, however the scale of change was varying a lot for different targets.

These results showed that when chemical libraries with compounds covering chemical space to quite high extent undergo virtual screening procedure, structures selected for training should also cover this space as much as possible. The other way, it seems to be the source of difficulties for machine learning methods to correctly identify potentially active ligands.

### Choosing the best machine learning method

The application of machine learning methods in virtual screening experiments is connected with the desire to correctly select potentially active compounds, also with structures that are different from the already known ligands. Commercially available databases that are evaluated in virtual screening procedure contain compounds with various structures and properties. Therefore, it is desirable that machine learning methods are able to correctly classify compounds that are different from those present in the training set.

Ibk was the method that was characterized by the highest efficiency in classification of compounds from the ZINC database, when trained on highly limited DUD sets and the ExtFP and MACCSFP were used as fingerprints (MCC ~0.2–0.3 for the former fingerprint and ~0.55–0.7 for the latter one). The SMO was the most effective, when molecules were represented by KlekFP with Ibk, Decorate and Random Forest only little worse comparing to them (MCC ~0.8–0.9). The last combination of learning algorithm-fingerprint enables the correct classification of molecules significantly different from those present in the training set.

Although the DUD sets are useful for docking studies, applying it for virtual screening experiments with the use of machine learning is not as effective. The high limitation of chemical space is a source of difficulties for learning algorithms to proper discriminate actives from inactives, when large libraries containing a great variety of chemical structures are evaluated. However, when they are limited to some extent (for example by other filters), forming training sets from more narrow databases may lead to improvement in classification effectiveness.

## Conclusions

Various ways of selection of compounds, that are assumed to be inactive are applied in computational experiments. However, this step of training set formation may have a significant impact on the effectiveness of classification performed by machine learning methods. Although, the DUD sets are widely applied in docking experiments with a high-level results [15], they are not necessarily appropriate for application in virtual screening experiments with the use of machine learning. As libraries of commercially available compounds contain a big variety of structures, machine learning algorithms are unable to select correctly potentially active compounds, when they are trained on inactive molecules covering a chemical space only to a small extent as it is in case with DUD. Because of that, inactive compounds chosen for training set should be as representative as possible for the libraries that undergo screening. It is also worth noting that in the majority of studies connected with examining the machine learning methods performance, compounds covering training and test sets are selected in an analogous way. Results of such researches will not necessarily coincide with real experiment (DUD sets also provided the best results when tested on similarly generated compounds, however they did not led to effective classification of various compounds from large database, as ZINC). What is more, out of the group of tested fingerprints, KlekFP should be applied for molecules representation, as it provided the highest efficiency of classification of molecules different from those used for training a classifier. The same goes for Ibk and SMO when particular learning methods are taken into consideration.

## Methods

The protein targets chosen for the experiments were: cyclooxygenase-2 (COX-2), muscarinic receptor M_1_, protease HIV-1 (HIV PR), matrix metalloproteinase and receptor 5-HT_1A_. They were selected after analysing a set of papers concerning different aspects of machine learning methods tests [16-19]. We chose such targets that were appearing most frequently in this kind of comparative studies and for which a sufficient number of known ligands exist. All compounds showing activity towards them were extracted from the MDDR database [13] – they formed a class of positive learning examples. Sets of compounds assumed as inactive were selected out of the three already mentioned databases: ZINC, MDDR and libraries that were generated in the way similar to DUD.

### Libraries generation according to DUD approach

Libraries of compounds with physicochemical properties similar to active ligands, and at the same time with dissimilar topology were created as follows. For each active ligand and for each structure from the ZINC database the set of descriptors was calculated using tools provided by ChemAxon [20]: logP, molecular weight (MW), number of hydrogen bond acceptors (HBA), number of hydrogen bond donors (HBD) and number of rotatable bonds (rotB). Using an in-house script, for each active ligand, structures with the same number of HBA, HBD and rotB as well as with the logP and MW values differing by no more than 10% were selected out of the ZINC database (for HIV protease inhibitors those settings were more flexible due to small number of decoys selected: HBA, HBD and rotB +−2, MW and logP values +−20%). Then, the Daylight-type fingerprints were calculated by means of the RDKit software [21], and the set of compounds was restricted only to those with Tanimoto coefficient less than 0.7 to particular ligand. For each one, 36 decoys (with the lowest values of Tanimoto coefficient) were picked up and formed the described library (Table 2).

**Table 2:** Number of decoys selected for each target

**Target**	**Number of input ligands**	**Number of decoys**
COX-2	1126	39508
M_1_	1155	32511
HIV PR	1135	11113
Metalloproteinase	788	19868
5-HT_1A_	1101	38477

### Formation of the set of inactives

From each of the three considered libraries of compounds, structures forming the set of inactives were selected in two different ways: random selection and selection providing maximum diversity of chosen molecules (the module Library Design from Discovery Studio 2.5 [22]). Number of selected compounds as well as the number of actives forming each training/test set are presented in Table 3.

**Table 3:** Composition of training and test sets used in the experiments

**Protein target**	**ligands**	**MDDR activity index**	**Number of actives/number of inactives**	
			**Train set**	**Test set**
COX-2	inhibitors	78454	242/316	884/950
M_1_	agonists	09249	281/315	874/950
HIV PR	inhibitors	71523	203/350	932/1100
Metalloproteinase	inhibitors	78432	144/280	644/800
5-HT_1A_	agonists	06235	198/340	903/1050

All experiments were performed in two modes:

•With the use of one common test set, the same for each method of inactive molecules generation, where inactives were randomly selected out of the ZINC database – common-test set mode,

•With the use of test sets with inactives prepared in the analogous way as for training – various-test sets mode.

For each part of the study that used randomization, the procedure of inactive molecules selection was repeated 10 times (during both train and test set generation). There was only 1 iteration of experiments selected in a diverse way, as (due to the way an algorithm of diverse selection works) the same set of compounds from particular database is always picked up.

Although selecting inactives for common-test set mode favours the ZINC random training set, this type of experiment resembles to the greatest extent virtual screening tasks, where libraries that undergo the procedure contain very diverse compounds. However, in order to provide an independent validation, an external validation test set was provided with inactives selected from PubChem database [23]. However, not for all the targets, true inactives were available in sufficient number and therefore the experiments in such mode were performed only for M_1_ (AID: 628; 61476 cmds), metalloproteinase (AID: 618; 86197 cmds) and 5-HT_1A_ (AID: 567; 64559 cmds) preserving 10 times randomization.

### Molecules representation

For each structure in the dataset, three different types of fingerprints were generated with the use of the PaDEL-Descriptor software [24]: Extended Fingerprint (ExtFP, 1024 bits) [25], MACCS Fingerprint (MACCSFP, 166 bits) [26] and Klekota & Roth Fingerprint (KlekFP, 4860 bits) [27].

### Machine learning experiments

Seven machine learning methods were selected for the experiments: Naïve Bayes classifier [2], Sequential Minimal Optimization [28], Instance-Based Learning [16], Decorate [29,30], Hyperpipes [31], J48 [2] and Random Forest [32,33]. For some methods, a series of tests were performed with varying settings for different classifiers (Table 1). Algorithms’ implementations present in the WEKA package (version 3.6) [34] were used. All calculations were performed on Intel Core i7 CPU 3.00 GHz computer system with 24 GB RAM running a 64-bit Linux operating system.

### The evaluation of machine learning methods

For machine learning methods evaluation three parameters were used: recall – R (1), precision – P (2), and the Matthews Correlation Coefficient – MCC (3):

(1) $$R=\frac{\mathrm{TP}}{\mathrm{TP}+\mathrm{FN}}$$

(2) $$P=\frac{\mathrm{TP}}{\mathrm{TP}+\mathrm{FP}}$$

(3) $$\mathrm{MCC}=\frac{\mathrm{TP}\cdot \mathrm{TN}-\mathrm{FP}\cdot \mathrm{FN}}{\sqrt{\left(\mathrm{TP}+\mathrm{FP}\right)\cdot \left(\mathrm{TP}+\mathrm{FN}\right)\cdot \left(\mathrm{TN}+\mathrm{FP}\right)\cdot \left(\mathrm{TN}+\mathrm{FN}\right)}}$$

Recall measures the fraction of correctly labelled positive examples, precision describes the correctness of positive instances prediction, whereas MCC is a balanced measure of binary classification effectiveness, ranging from −1 to 1, where 1 corresponds to error-free class labelling and −1 to reverse classification [7,35].

## Competing interests

The authors declare that they have no competing interests.

## Authors' contributions

All authors designed the experiments. SS and RK performed the experiments. All authors analyzed the data and draw conclusions and read and approved the final manuscript.

## Supplementary Material

Additional file 1: Figure S1 ML methods performance for various parameters in classification of metalloproteinase inhibitors. **Figure S1** presents an exemplary panel of values of evaluating parameters obtained for various parameters of machine learning methods. Click here for file

Additional file 2: Tables S1-S3 Numerical values of evaluating parameters values obtained in the common-test set mode (**Table S1**), various-test set mode (**Table S2**) and their standard deviations (**Table S3**). Click here for file

Additional file 3: Figure S2 A a panel of heat maps obtained in tests with external sets of in actives fetched from PubChem database. Click here for file

Additional file 4: Tables S4 Numerical values of evaluating parameters obtained in experiments with inactives from PubChem database. Click here for file
