# Supplementary material for: The influence of the inactives subset generation on the performance of machine learning methods
Source: J Cheminform. 2013 Apr 5;5:17. doi: 10.1186/1758-2946-5-17 (PMC3626618; doi:10.1186/1758-2946-5-17)
Supplement: Additional file 1: Figure S1 — ML methods performance for various parameters in classification of metalloproteinase inhibitors. Figure S1 presents an exemplary panel of values of evaluating parameters obtained for various parameters of machine learning methods. [file 1758-2946-5-17-S1.pdf]

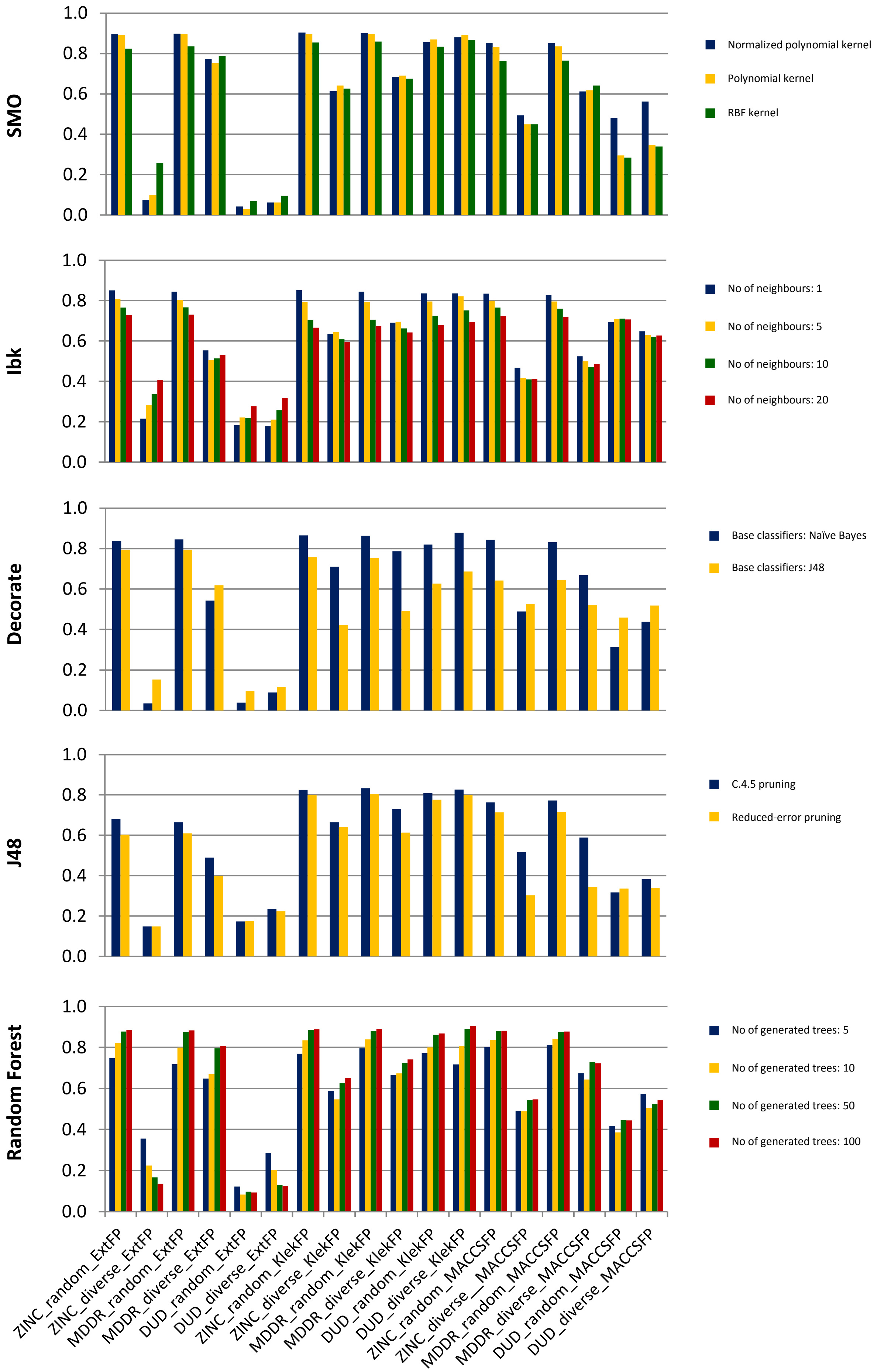

Figure S1. ML methods performance for various parameters in classification of metalloproteinase inhibitors
