# Supplementary material for: The influence of the inactives subset generation on the performance of machine learning methods
Source: J Cheminform. 2013 Apr 5;5:17. doi: 10.1186/1758-2946-5-17 (PMC3626618; doi:10.1186/1758-2946-5-17)
Supplement: Additional file 2: Tables S1-S3 — Numerical values of evaluating parameters values obtained in the common-test set mode (Table S1), various-test set mode (Table S2) and their standard deviations (Table S3). [file 1758-2946-5-17-S2.pdf]

Table S1. Evaluating parameters values obtained in the common-test set mode

COX-2 (common-test set mode)										
**ML method**	**inactives selection**	**ExtFP**			**KlekFP**			**MACCSFP**		
		**recall**	**precision**	**MCC**	**recall**	**precision**	**MCC**	**recall**	**precision**	**MCC**
Naïve Bayes	ZINC_random	**0.95**	**0.97**	**0.92**	**0.98**	**0.93**	**0.91**	**0.98**	**0.95**	**0.93**
	ZINC_diverse	**0.99**	0.79	0.75	**0.98**	0.88	0.86	**0.97**	0.88	0.85
	MDDR_random	**0.94**	**0.99**	**0.93**	**0.98**	**0.91**	0.89	**0.97**	**0.92**	0.89
	MDDR_diverse	**0.93**	**0.99**	**0.92**	**0.98**	**0.93**	**0.91**	**0.97**	0.89	0.86
	DUD_random	**1**	0.56	0.37	**0.99**	**0.91**	0.9	**1**	0.81	0.8
	DUD_diverse	**0.99**	0.66	0.58	**0.99**	**0.97**	**0.96**	**0.99**	0.85	0.83
SMO	ZINC_random	**1**	**1**	**1**	**0.99**	**1**	**0.99**	**0.99**	**1**	**0.99**
	ZINC_diverse	**1**	0.54	0.33	**0.99**	**1**	**0.99**	**0.99**	**0.95**	**0.94**
	MDDR_random	**1**	**1**	**1**	**0.99**	**1**	**0.99**	**0.98**	**1**	**0.98**
	MDDR_diverse	**1**	**1**	**1**	**0.99**	**1**	**0.99**	**0.99**	**1**	**0.98**
	DUD_random	**1**	0.52	0.29	**0.99**	**1**	**0.99**	**1**	0.85	0.85
	DUD_diverse	**1**	0.54	0.33	**0.99**	**1**	**0.99**	**1**	**0.93**	**0.92**
Ibk	ZINC_random	**1**	**0.99**	**0.99**	**0.95**	**0.99**	**0.94**	**0.97**	**0.99**	**0.96**
	ZINC_diverse	**1**	0.77	0.75	**0.93**	**0.98**	**0.91**	**0.98**	0.88	0.85
	MDDR_random	**0.99**	**0.99**	**0.98**	**0.92**	**0.99**	**0.91**	**0.96**	**0.97**	**0.94**
	MDDR_diverse	**0.98**	**0.98**	**0.96**	**0.91**	**0.99**	**0.91**	**0.97**	**0.91**	0.88
	DUD_random	**1**	0.74	0.71	**0.94**	**1**	**0.94**	**0.99**	**0.94**	**0.93**
	DUD_diverse	**1**	0.77	0.75	**0.91**	**1**	**0.92**	**0.99**	**0.93**	**0.92**
Decorate	ZINC_random	**1**	**1**	**0.99**	**0.98**	**1**	**0.98**	**0.99**	**1**	**0.99**
	ZINC_diverse	**1**	0.61	0.49	**0.97**	**1**	**0.97**	**0.97**	**0.98**	**0.96**
	MDDR_random	**1**	**1**	**0.99**	**0.99**	**1**	**0.99**	**0.99**	**1**	**0.99**
	MDDR_diverse	**0.99**	**1**	**0.99**	**0.98**	**1**	**0.98**	**0.99**	**1**	**0.99**
	DUD_random	**1**	0.66	0.57	**0.98**	**1**	**0.98**	**1**	0.61	0.49
	DUD_diverse	**1**	0.57	0.42	**0.98**	**1**	**0.98**	**0.99**	0.82	0.8
Hyperpipes	ZINC_random	**1**	**0.97**	**0.97**	**1**	0.86	0.85	**1**	0.63	0.53
	ZINC_diverse	**1**	0.88	0.87	**1**	0.76	0.74	**0.99**	0.62	0.51
	MDDR_random	**1**	**0.97**	**0.97**	**1**	0.84	0.83	**1**	0.63	0.53
	MDDR_diverse	**1**	**0.97**	**0.97**	**1**	0.81	0.79	**1**	0.63	0.53
	DUD_random	**1**	0.87	0.87	**1**	0.73	0.7	**1**	0.58	0.44
	DUD_diverse	**1**	0.88	0.87	**1**	0.77	0.75	**1**	0.6	0.48
J48	ZINC_random	**0.99**	**0.96**	**0.95**	**0.96**	**0.99**	**0.95**	**0.99**	**1**	**0.98**
	ZINC_diverse	**1**	0.83	0.82	**0.96**	**1**	**0.97**	**0.97**	**0.99**	**0.96**
	MDDR_random	**0.99**	**0.98**	**0.97**	**0.98**	**1**	**0.98**	**0.99**	**1**	**0.99**
	MDDR_diverse	**0.97**	**0.99**	**0.96**	**0.96**	**1**	**0.96**	**0.98**	**1**	**0.98**
	DUD_random	**1**	0.71	0.66	**0.96**	**0.99**	**0.96**	**1**	0.6	0.48
	DUD_diverse	**1**	0.53	0.32	**0.96**	**1**	**0.97**	**1**	0.87	0.86
Random Forest	ZINC_random	**0.99**	**0.99**	**0.99**	**0.98**	**1**	**0.98**	**0.99**	**1**	**0.99**
	ZINC_diverse	**1**	0.75	0.72	**0.97**	**0.98**	**0.95**	**0.98**	**0.92**	**0.91**
	MDDR_random	**0.99**	**1**	**0.99**	**0.97**	**1**	**0.97**	**0.98**	**1**	**0.98**
	MDDR_diverse	**0.98**	**1**	**0.98**	**0.98**	**0.99**	**0.97**	**0.99**	**0.97**	**0.95**
	DUD_random	**1**	0.61	0.5	**0.99**	**1**	**0.98**	**1**	0.71	0.67
	DUD_diverse	**1**	0.75	0.72	**0.97**	**1**	**0.97**	**1**	0.9	0.89

M_1 (common-test set mode)										
**ML method**	**inactives selection**	**ExtFP**			**KlekFP**			**MACCSFP**		
		**recall**	**precision**	**MCC**	**recall**	**precision**	**MCC**	**recall**	**precision**	**MCC**
Naïve Bayes	ZINC_random	0.67	0.9	0.59	0.81	**0.95**	0.78	0.87	0.77	0.64
	ZINC_diverse	0.7	0.67	0.37	**0.91**	0.66	0.5	0.85	0.68	0.49
	MDDR_random	0.74	0.65	0.44	0.87	0.86	0.76	0.76	0.82	0.64
	MDDR_diverse	**0.96**	0.54	0.28	0.89	0.8	0.69	0.89	0.64	0.46
	DUD_random	**0.93**	0.65	0.5	0.9	0.8	0.7	0.9	0.72	0.58
	DUD_diverse	0.76	0.86	0.65	**0.91**	0.79	0.69	0.84	0.73	0.56
SMO	ZINC_random	**0.93**	**0.97**	0.89	**0.95**	**0.93**	0.88	**0.96**	0.81	0.76
	ZINC_diverse	**0.98**	0.68	0.68	**0.98**	0.6	0.46	**0.96**	0.66	0.54
	MDDR_random	0.9	**0.96**	0.84	**0.94**	**0.95**	0.9	**0.91**	**0.92**	0.85
	MDDR_diverse	**1**	0.48	0.06	**0.96**	0.71	0.63	**0.95**	0.53	0.24
	DUD_random	**0.98**	0.74	0.68	**0.96**	0.86	0.82	**0.94**	0.81	0.74
	DUD_diverse	**0.96**	0.73	0.65	**0.94**	0.79	0.72	**0.93**	0.79	0.71
Ibk	ZINC_random	**0.99**	0.84	0.83	**0.98**	0.81	0.78	**0.99**	0.75	0.71
	ZINC_diverse	**0.99**	0.58	0.61	**0.99**	0.62	0.51	**0.99**	0.57	0.41
	MDDR_random	**0.97**	0.43	0.55	**0.93**	0.9	0.84	**0.94**	0.87	0.83
	MDDR_diverse	**1**	0.51	0.24	**0.97**	0.66	0.56	**0.99**	0.55	0.37
	DUD_random	**0.96**	0.84	0.79	**0.98**	0.78	0.74	**0.98**	0.75	0.69
	DUD_diverse	**0.96**	0.71	0.63	**0.98**	0.75	0.69	**0.98**	0.7	0.62
Decorate	ZINC_random	0.77	0.86	0.64	**0.92**	**0.93**	0.86	**0.94**	0.78	0.7
	ZINC_diverse	0.7	0.67	0.37	**0.97**	0.64	0.53	**0.95**	0.63	0.48
	MDDR_random	0.78	0.77	0.42	0.89	**0.95**	0.86	0.88	**0.93**	0.83
	MDDR_diverse	**1**	0.48	0.07	**0.92**	0.79	0.7	**0.94**	0.53	0.26
	DUD_random	**0.96**	0.71	0.62	**0.93**	0.84	0.77	**0.93**	0.78	0.69
	DUD_diverse	**0.95**	0.71	0.62	**0.94**	0.82	0.75	0.9	0.8	0.69
Hyperpipes	ZINC_random	**0.99**	0.49	0.54	0.86	0.77	0.63	**0.99**	0.49	0.13
	ZINC_diverse	**0.98**	0.49	0.53	**0.91**	0.67	0.52	**0.99**	0.49	0.13
	MDDR_random	**0.98**	0.37	0.08	**0.93**	0.7	0.62	**0.99**	0.48	0.28
	MDDR_diverse	**0.98**	0.49	0.08	0.87	0.7	0.54	**0.98**	0.49	0.12
	DUD_random	**0.99**	0.49	0.1	0.89	0.66	0.49	**0.99**	0.49	0.1
	DUD_diverse	**0.98**	0.49	0.08	**0.92**	0.69	0.57	**0.99**	0.49	0.13
J48	ZINC_random	0.85	0.86	0.7	0.89	0.89	0.79	**0.92**	0.75	0.64
	ZINC_diverse	**0.92**	0.59	0.54	**0.96**	0.62	0.48	**0.93**	0.74	0.64
	MDDR_random	0.86	0.85	0.65	0.87	**0.93**	0.83	0.85	0.89	0.77
	MDDR_diverse	**0.99**	0.49	0.13	0.9	0.76	0.65	**0.94**	0.5	0.12
	DUD_random	0.89	0.7	0.55	0.89	0.78	0.66	0.89	0.77	0.65
	DUD_diverse	0.9	0.7	0.56	0.9	0.81	0.7	0.86	0.79	0.66
Random Forest	ZINC_random	**0.93**	**0.97**	0.9	**0.94**	**0.95**	0.89	**0.97**	0.82	0.78
	ZINC_diverse	**0.97**	0.7	0.68	**0.98**	0.69	0.61	**0.99**	0.68	0.61
	MDDR_random	**0.91**	**0.96**	0.85	**0.91**	**0.97**	0.89	0.9	**0.96**	0.88
	MDDR_diverse	**1**	0.49	0.11	**0.96**	0.77	0.7	**0.96**	0.58	0.4
	DUD_random	**0.97**	0.77	0.72	**0.96**	0.87	0.82	**0.95**	0.84	0.79
	DUD_diverse	**0.96**	0.77	0.71	**0.96**	0.86	0.82	**0.94**	0.85	0.78

HIV PR (common-test set mode)										
**ML method**	**inactives selection**	**ExtFP**			**KlekFP**			**MACCSFP**		
		**recall**	**precision**	**MCC**	**recall**	**precision**	**MCC**	**recall**	**precision**	**MCC**
Naïve Bayes	ZINC_random	**0.91**	**0.96**	0.89	**0.93**	**0.93**	0.87	0.85	0.88	0.76
	ZINC_diverse	**0.98**	0.61	0.51	**0.96**	0.67	0.59	**0.96**	0.66	0.57
	MDDR_random	0.85	**0.97**	0.84	0.86	**0.92**	0.81	0.76	0.83	0.63
	MDDR_diverse	**0.92**	**0.91**	0.84	**0.92**	0.73	0.64	0.89	0.7	0.58
	DUD_random	**0.98**	0.59	0.37	**0.96**	0.72	0.64	**0.99**	0.54	0.35
	DUD_diverse	**0.99**	0.49	0.23	**0.92**	0.79	0.71	**0.95**	0.6	0.45
SMO	ZINC_random	**0.99**	**0.97**	**0.97**	**0.98**	**0.97**	**0.96**	**0.95**	**0.95**	**0.91**
	ZINC_diverse	**1**	0.48	0.21	**0.99**	0.79	0.77	**0.99**	0.63	0.55
	MDDR_random	**0.95**	**0.97**	**0.92**	**0.96**	**0.94**	**0.91**	0.89	0.88	0.78
	MDDR_diverse	**0.97**	0.87	0.84	**0.97**	0.85	0.82	**0.95**	0.79	0.74
	DUD_random	**0.99**	0.61	0.38	**0.99**	0.76	0.68	**1**	0.54	0.34
	DUD_diverse	**1**	0.47	0.14	**0.97**	**0.91**	0.88	**0.99**	0.65	0.59
Ibk	ZINC_random	**0.99**	**0.96**	**0.95**	**0.98**	**0.94**	**0.93**	**0.97**	0.87	0.85
	ZINC_diverse	**1**	0.56	0.43	**0.99**	0.8	0.78	**1**	0.58	0.48
	MDDR_random	**0.98**	**0.92**	**0.91**	**0.97**	0.89	0.87	**0.96**	0.74	0.69
	MDDR_diverse	**0.99**	0.83	0.83	**0.98**	0.84	0.82	**0.98**	0.62	0.52
	DUD_random	**0.99**	0.68	0.58	**0.99**	0.84	0.81	**0.99**	0.63	0.54
	DUD_diverse	**1**	0.56	0.43	**0.98**	**0.92**	**0.91**	**0.99**	0.65	0.59
Decorate	ZINC_random	**0.95**	**0.97**	**0.92**	**0.95**	**0.96**	**0.92**	**0.91**	**0.94**	0.87
	ZINC_diverse	**1**	0.48	0.2	**0.98**	0.81	0.79	**0.99**	0.65	0.58
	MDDR_random	**0.91**	**0.97**	0.89	**0.93**	**0.96**	0.9	0.88	0.88	0.78
	MDDR_diverse	**0.97**	**0.94**	**0.91**	**0.96**	0.88	0.84	**0.93**	0.78	0.7
	DUD_random	**0.99**	0.62	0.42	**0.97**	0.78	0.68	**1**	0.52	0.31
	DUD_diverse	**0.99**	0.55	0.39	**0.96**	**0.91**	0.88	**1**	0.54	0.38
Hyperpipes	ZINC_random	**1**	0.46	0.1	0.74	0.74	0.64	**0.99**	0.51	0.29
	ZINC_diverse	**1**	0.46	0.06	**0.95**	0.64	0.53	**0.98**	0.51	0.28
	MDDR_random	**1**	0.47	0.11	0.86	0.7	0.55	**0.98**	0.51	0.28
	MDDR_diverse	**1**	0.47	0.11	0.89	0.67	0.52	**0.98**	0.51	0.28
	DUD_random	**0.98**	0.51	0.2	**0.97**	0.56	0.4	**1**	0.49	0.22
	DUD_diverse	**1**	0.46	0.06	**0.94**	0.64	0.51	**0.99**	0.51	0.3
J48	ZINC_random	**0.93**	0.9	0.84	**0.94**	**0.94**	0.88	0.88	0.9	0.8
	ZINC_diverse	**1**	0.46	0.09	**0.95**	0.83	0.79	**0.98**	0.68	0.61
	MDDR_random	0.86	**0.91**	0.79	0.9	**0.94**	0.86	0.84	0.82	0.68
	MDDR_diverse	**0.92**	0.8	0.72	**0.94**	0.86	0.81	**0.95**	0.67	0.58
	DUD_random	**0.98**	0.64	0.48	**0.94**	0.76	0.64	**0.99**	0.49	0.24
	DUD_diverse	**0.99**	0.61	0.52	**0.94**	**0.91**	0.86	**0.98**	0.56	0.41
Random Forest	ZINC_random	**0.95**	**0.99**	**0.95**	**0.97**	**0.99**	**0.96**	**0.95**	**0.97**	**0.93**
	ZINC_diverse	**1**	0.56	0.44	**0.98**	0.85	0.84	**1**	0.66	0.6
	MDDR_random	0.9	**1**	**0.91**	**0.95**	**1**	**0.95**	**0.92**	**0.95**	0.88
	MDDR_diverse	**0.97**	**0.97**	**0.95**	**0.96**	**0.92**	0.89	**0.97**	0.8	0.77
	DUD_random	**0.99**	0.64	0.47	**0.98**	0.82	0.75	**1**	0.53	0.34
	DUD_diverse	**1**	0.51	0.29	**0.96**	**0.99**	**0.96**	**0.99**	0.59	0.48

metalloproteinase (common-test set mode)											
ML method	inactives selection	ExtFP			KlekFP			MACCSFP			
		recall	precision	MCC	recall	precision	MCC	recall	precision	MCC	
Naïve Bayes	ZINC_random	0.82	**0.94**	0.79	0.87	0.87	0.76	0.76	0.83	0.64	
	ZINC_diverse	**0.97**	0.47	0.15	**0.95**	0.57	0.42	**0.95**	0.63	0.53	
	MDDR_random	0.83	**0.93**	0.79	0.87	0.86	0.76	0.76	0.82	0.64	
	MDDR_diverse	0.89	0.72	0.62	**0.92**	0.62	0.49	0.88	0.66	0.52	
	DUD_random	**1**	0.45	0.1	0.88	0.79	0.69	**0.99**	0.57	0.46	
	DUD_diverse	**0.99**	0.46	0.12	0.89	0.8	0.71	**0.96**	0.62	0.52	
SMO	ZINC_random	**0.91**	**0.97**	0.9	**0.94**	**0.95**	0.9	**0.91**	**0.93**	0.85	
	ZINC_diverse	**1**	0.45	0.07	**0.97**	0.67	0.61	**0.98**	0.59	0.49	
	MDDR_random	**0.92**	**0.97**	0.9	**0.94**	**0.95**	0.9	**0.91**	**0.92**	0.85	
	MDDR_diverse	**0.93**	0.83	0.77	**0.95**	0.74	0.68	**0.93**	0.7	0.61	
	DUD_random	**1**	0.45	0.04	**0.95**	0.89	0.86	**0.98**	0.59	0.48	
	DUD_diverse	**1**	0.45	0.06	**0.95**	**0.92**	0.88	**0.98**	0.64	0.56	
Ibk	ZINC_random	**0.93**	0.9	0.85	**0.93**	**0.91**	0.85	**0.94**	0.88	0.83	
	ZINC_diverse	**0.99**	0.48	0.21	**0.97**	0.69	0.64	**0.99**	0.57	0.47	
	MDDR_random	**0.94**	0.89	0.84	**0.93**	0.9	0.84	**0.94**	0.87	0.83	
	MDDR_diverse	**0.95**	0.64	0.55	**0.94**	0.75	0.69	**0.97**	0.61	0.52	
	DUD_random	**0.99**	0.47	0.18	**0.94**	0.88	0.84	**0.98**	0.73	0.69	
	DUD_diverse	**0.99**	0.47	0.18	**0.94**	0.88	0.84	**0.99**	0.69	0.65	
Decorate	ZINC_random	0.87	**0.94**	0.84	0.9	**0.95**	0.86	0.88	**0.94**	0.84	
	ZINC_diverse	**1**	0.45	0.04	**0.95**	0.76	0.71	**0.96**	0.6	0.49	
	MDDR_random	0.89	**0.94**	0.85	0.89	**0.95**	0.86	0.88	**0.93**	0.83	
	MDDR_diverse	**0.91**	0.66	0.54	**0.92**	0.85	0.79	**0.94**	0.74	0.67	
	DUD_random	**1**	0.45	0.04	0.9	0.9	0.82	**0.99**	0.51	0.31	
	DUD_diverse	**1**	0.45	0.09	**0.92**	**0.94**	0.88	**1**	0.55	0.44	
Hyperpipes	ZINC_random	**1**	0.45	0.04	**0.93**	0.71	0.62	**0.99**	0.49	0.28	
	ZINC_diverse	**0.99**	0.45	0	**0.98**	0.59	0.49	**0.98**	0.49	0.25	
	MDDR_random	**1**	0.44	0.04	**0.93**	0.7	0.62	**0.99**	0.48	0.28	
	MDDR_diverse	**0.99**	0.45	0.04	0.88	0.61	0.44	**0.98**	0.49	0.25	
	DUD_random	**1**	0.45	0	**0.97**	0.63	0.54	**0.99**	0.49	0.27	
	DUD_diverse	**0.99**	0.45	0	**0.95**	0.66	0.57	**0.99**	0.49	0.27	
J48	ZINC_random	0.82	0.82	0.68	0.87	**0.93**	0.82	0.84	0.89	0.76	
	ZINC_diverse	**0.99**	0.46	0.15	**0.94**	0.73	0.67	**0.94**	0.63	0.52	
	MDDR_random	0.81	0.81	0.66	0.87	**0.93**	0.83	0.85	0.89	0.77	
	MDDR_diverse	0.87	0.64	0.49	**0.92**	0.8	0.73	0.89	0.7	0.59	
	DUD_random	**0.99**	0.47	0.17	0.88	0.9	0.81	**0.96**	0.52	0.32	
	DUD_diverse	**0.99**	0.48	0.23	0.9	**0.91**	0.83	**0.97**	0.54	0.38	
Random Forest	ZINC_random	0.87	**0.99**	0.88	0.9	**0.97**	0.89	0.9	**0.97**	0.88	
	ZINC_diverse	**0.99**	0.46	0.13	**0.96**	0.71	0.65	**0.98**	0.62	0.55	
	MDDR_random	0.88	**0.99**	0.88	**0.91**	**0.97**	0.89	0.9	**0.96**	0.88	
	MDDR_diverse	**0.91**	0.88	0.81	**0.96**	0.78	0.74	**0.95**	0.77	0.72	
	DUD_random	**1**	0.45	0.09	**0.92**	**0.93**	0.87	**0.99**	0.56	0.44	
	DUD_diverse	**1**	0.45	0.12	**0.93**	**0.97**	0.9	**1**	0.61	0.54	

5-HT_{1A} (common-test set mode)										
**ML method**	**inactives selection**	**ExtFP**			**KlekFP**			**MACCSFP**		
		**recall**	**precision**	**MCC**	**recall**	**precision**	**MCC**	**recall**	**precision**	**MCC**
Naïve Bayes	ZINC_random	0.86	0.86	0.74	0.88	0.9	0.79	0.87	0.79	0.67
	ZINC_diverse	**0.99**	0.49	0.19	**0.95**	0.57	0.4	**0.92**	0.61	0.44
	MDDR_random	0.88	0.6	0.4	0.89	0.83	0.73	0.83	0.74	0.58
	MDDR_diverse	**0.94**	0.66	0.56	**0.93**	0.64	0.51	**0.92**	0.63	0.49
	DUD_random	**1**	0.47	0.11	**0.91**	0.68	0.56	**0.95**	0.55	0.36
	DUD_diverse	**1**	0.47	0.12	**0.91**	0.64	0.5	**0.95**	0.59	0.43
SMO	ZINC_random	**0.95**	**0.95**	**0.91**	**0.94**	**0.92**	0.87	**0.93**	**0.92**	0.86
	ZINC_diverse	**1**	0.47	0.11	**0.96**	0.66	0.57	**0.99**	0.58	0.46
	MDDR_random	**0.92**	**0.91**	0.84	**0.92**	0.88	0.8	0.84	0.78	0.64
	MDDR_diverse	**0.95**	0.81	0.76	**0.94**	0.72	0.65	**0.92**	0.7	0.59
	DUD_random	**1**	0.47	0.08	**0.95**	0.86	0.81	**1**	0.56	0.44
	DUD_diverse	**1**	0.47	0.1	**0.94**	0.84	0.78	**0.99**	0.6	0.5
Ibk	ZINC_random	**0.96**	0.86	0.83	**0.95**	0.84	0.8	**0.96**	0.85	0.81
	ZINC_diverse	**1**	0.51	0.28	**0.97**	0.66	0.58	**1**	0.56	0.43
	MDDR_random	**0.97**	0.71	0.64	**0.94**	0.76	0.69	**0.94**	0.73	0.65
	MDDR_diverse	**0.97**	0.62	0.52	**0.94**	0.71	0.63	**0.97**	0.62	0.52
	DUD_random	**1**	0.5	0.25	**0.96**	0.77	0.72	**0.98**	0.66	0.59
	DUD_diverse	**1**	0.5	0.27	**0.94**	0.78	0.71	**0.99**	0.63	0.56
Decorate	ZINC_random	0.87	**0.92**	0.81	0.9	**0.92**	0.84	**0.92**	**0.94**	0.86
	ZINC_diverse	**1**	0.47	0.11	**0.96**	0.67	0.58	**0.99**	0.6	0.5
	MDDR_random	0.86	0.88	0.76	0.89	0.88	0.79	0.85	0.83	0.7
	MDDR_diverse	**0.93**	0.72	0.63	**0.94**	0.76	0.69	0.88	0.74	0.61
	DUD_random	**1**	0.47	0.09	**0.94**	0.82	0.77	**1**	0.51	0.3
	DUD_diverse	**1**	0.47	0.1	0.9	0.83	0.74	**1**	0.54	0.38
Hyperpipes	ZINC_random	**1**	0.5	0.25	0.87	0.81	0.69	**0.99**	0.52	0.31
	ZINC_diverse	**1**	0.48	0.19	**0.91**	0.7	0.59	**0.99**	0.52	0.32
	MDDR_random	**1**	0.5	0.25	0.84	0.78	0.63	**0.99**	0.52	0.31
	MDDR_diverse	**1**	0.5	0.25	0.87	0.72	0.58	**0.99**	0.52	0.31
	DUD_random	**1**	0.48	0.19	**0.91**	0.68	0.56	**1**	0.52	0.31
	DUD_diverse	**1**	0.48	0.19	0.9	0.72	0.6	**1**	0.52	0.31
J48	ZINC_random	0.81	0.84	0.68	0.87	0.89	0.78	0.88	**0.91**	0.81
	ZINC_diverse	**0.99**	0.47	0.08	**0.92**	0.65	0.51	**0.99**	0.63	0.54
	MDDR_random	0.77	0.75	0.54	0.86	0.85	0.73	0.82	0.77	0.6
	MDDR_diverse	0.85	0.71	0.55	0.9	0.68	0.55	0.89	0.68	0.54
	DUD_random	**0.99**	0.48	0.14	0.88	0.81	0.71	**0.99**	0.51	0.29
	DUD_diverse	**1**	0.47	0.13	**0.94**	0.75	0.67	**1**	0.52	0.32
Random Forest	ZINC_random	0.88	**0.98**	0.87	**0.92**	**0.95**	0.88	**0.94**	**0.95**	0.89
	ZINC_diverse	**1**	0.48	0.17	**0.97**	0.69	0.62	**0.99**	0.65	0.59
	MDDR_random	0.86	**0.96**	0.83	**0.91**	**0.93**	0.85	0.88	0.9	0.8
	MDDR_diverse	**0.94**	0.81	0.76	**0.95**	0.78	0.73	**0.94**	0.77	0.7
	DUD_random	**1**	0.47	0.12	**0.94**	0.85	0.8	**0.99**	0.57	0.43
	DUD_diverse	**1**	0.48	0.15	**0.94**	0.87	0.81	**0.99**	0.6	0.49

Table S2. Evaluating parameters values obtained in the various-test set mode

COX-2 (various-test sets mode)										
**ML method**	**inactives selection**	**ExtFP**			**KlekFP**			**MACCSFP**		
		**recall**	**precision**	**MCC**	**recall**	**precision**	**MCC**	**recall**	**precision**	**MCC**
Naïve Bayes	ZINC_random	**0.95**	**0.96**	**0.92**	**0.98**	**0.92**	0.9	**0.98**	**0.95**	**0.93**
	ZINC_diverse	**0.99**	0.78	0.75	**0.98**	**0.94**	**0.92**	**0.97**	**0.95**	**0.92**
	MDDR_random	**0.93**	**0.93**	0.87	**0.98**	0.87	0.85	**0.97**	**0.94**	0.9
	MDDR_diverse	**0.93**	**0.91**	0.85	**0.98**	**0.92**	0.9	**0.97**	**0.92**	0.88
	DUD_random	**1**	**1**	**0.99**	**0.99**	**0.92**	**0.91**	**1**	**1**	**1**
	DUD_diverse	**0.99**	**1**	**0.99**	**0.99**	**0.96**	**0.92**	**0.99**	**0.99**	**0.97**
SMO	ZINC_random	**1**	**1**	**1**	**0.99**	**1**	**0.99**	**0.99**	**1**	**0.98**
	ZINC_diverse	**1**	0.7	0.65	**0.99**	**1**	**0.99**	**0.99**	**0.95**	**0.94**
	MDDR_random	**1**	**1**	**1**	**0.99**	**1**	**0.99**	**0.98**	**1**	**0.98**
	MDDR_diverse	**1**	**1**	**1**	**0.99**	**1**	**0.99**	**0.99**	**1**	**0.99**
	DUD_random	**1**	**1**	**1**	**0.99**	**1**	**0.99**	**1**	**1**	**1**
	DUD_diverse	**1**	**1**	**1**	**0.99**	**1**	**0.98**	**1**	**1**	**1**
Ibk	ZINC_random	**1**	**0.99**	**0.99**	**0.95**	**0.99**	**0.94**	**0.97**	**0.98**	**0.96**
	ZINC_diverse	**1**	0.77	0.75	**0.93**	**0.99**	**0.92**	**0.98**	**0.92**	0.9
	MDDR_random	**0.99**	**0.97**	**0.96**	**0.92**	**0.99**	**0.91**	**0.96**	**0.97**	**0.93**
	MDDR_diverse	**0.99**	**0.95**	**0.95**	**0.91**	**0.99**	**0.91**	**0.97**	**0.93**	**0.91**
	DUD_random	**1**	**1**	**1**	**0.94**	**0.99**	**0.94**	**0.99**	**1**	**0.99**
	DUD_diverse	**1**	**1**	**1**	**0.92**	**0.99**	0.88	**0.99**	**0.99**	**0.97**
Decorate	ZINC_random	**1**	**1**	**0.99**	**0.98**	**1**	**0.98**	**0.99**	**1**	**0.99**
	ZINC_diverse	**1**	0.7	0.65	**0.97**	**1**	**0.97**	**0.97**	**0.99**	**0.97**
	MDDR_random	**1**	**0.99**	**0.99**	**0.99**	**1**	**0.99**	**0.99**	**1**	**0.98**
	MDDR_diverse	**0.99**	**0.99**	**0.98**	**0.98**	**1**	**0.98**	**0.99**	**1**	**0.99**
	DUD_random	**1**	**1**	**1**	**0.98**	**1**	**0.98**	**1**	**0.99**	**0.99**
	DUD_diverse	**1**	**1**	**1**	**0.98**	**1**	**0.97**	**1**	**0.99**	**0.98**
Hyperpipes	ZINC_random	**1**	**0.97**	**0.97**	**1**	0.86	0.85	**1**	0.63	0.53
	ZINC_diverse	**1**	0.87	0.86	**1**	0.73	0.69	**0.99**	0.73	0.68
	MDDR_random	**1**	**0.95**	**0.95**	**1**	0.81	0.8	**1**	0.64	0.55
	MDDR_diverse	**1**	**0.98**	**0.98**	**1**	0.72	0.68	**1**	0.68	0.62
	DUD_random	**1**	**1**	**1**	**1**	0.85	0.84	**1**	**0.99**	**0.99**
	DUD_diverse	**1**	**1**	**1**	**1**	0.81	0.67	**1**	**0.96**	**0.94**
J48	ZINC_random	**0.99**	**0.96**	**0.95**	**0.96**	**0.99**	**0.95**	**0.99**	**1**	**0.98**
	ZINC_diverse	**1**	0.78	0.75	**0.96**	**1**	**0.97**	**0.97**	**0.97**	**0.94**
	MDDR_random	**0.99**	**0.98**	**0.97**	**0.98**	**1**	**0.97**	**0.99**	**0.99**	**0.98**
	MDDR_diverse	**0.98**	**0.97**	**0.95**	**0.96**	**1**	**0.96**	**0.98**	**1**	**0.98**
	DUD_random	**0.99**	**0.99**	**0.99**	**0.96**	**1**	**0.96**	**1**	**0.99**	**0.99**
	DUD_diverse	**0.99**	**0.99**	**0.97**	**0.97**	**1**	**0.96**	**0.99**	**0.97**	**0.95**
Random Forest	ZINC_random	**1**	**1**	**0.99**	**0.98**	**1**	**0.98**	**0.99**	**1**	**0.99**
	ZINC_diverse	**1**	0.82	0.8	**0.97**	**1**	**0.97**	**0.98**	**0.97**	**0.95**
	MDDR_random	**0.99**	**0.99**	**0.99**	**0.97**	**1**	**0.97**	**0.98**	**1**	**0.98**
	MDDR_diverse	**0.99**	**0.99**	**0.99**	**0.98**	**0.99**	**0.97**	**0.99**	**0.98**	**0.97**
	DUD_random	**1**	**1**	**1**	**0.98**	**1**	**0.98**	**1**	**1**	**1**
	DUD_diverse	**1**	**1**	**1**	**0.98**	**0.99**	**0.96**	**1**	**0.99**	**0.98**

M_1 (various-test sets mode)										
**ML method**	**inactives selection**	**ExtFP**			**KlekFP**			**MACCSFP**		
		**recall**	**precision**	**MCC**	**recall**	**precision**	**MCC**	**recall**	**precision**	**MCC**
Naïve Bayes	ZINC_random	0.66	0.89	0.57	0.81	**0.91**	0.73	0.78	0.88	0.66
	ZINC_diverse	0.74	**0.91**	0.66	**0.91**	0.9	0.82	0.85	**0.97**	0.84
	MDDR_random	0.74	0.79	0.53	0.8	0.87	0.67	0.76	0.79	0.55
	MDDR_diverse	0.62	0.83	0.45	0.89	0.88	0.77	0.85	0.78	0.63
	DUD_random	**0.92**	**0.91**	0.84	0.9	**0.91**	0.82	0.9	0.78	0.67
	DUD_diverse	0.76	**0.93**	0.72	**0.91**	0.88	0.79	0.84	0.74	0.57
SMO	ZINC_random	**0.93**	**0.97**	0.9	**0.91**	**0.96**	0.87	0.9	**0.95**	0.85
	ZINC_diverse	**0.98**	**1**	**0.98**	**0.98**	**0.92**	0.9	**0.96**	**0.95**	**0.92**
	MDDR_random	0.9	**0.95**	0.85	**0.93**	**0.91**	0.83	0.84	0.89	0.74
	MDDR_diverse	**0.94**	**0.93**	0.87	**0.95**	0.9	0.85	0.9	0.86	0.76
	DUD_random	**0.98**	**0.95**	**0.93**	**0.96**	**0.94**	0.9	**0.94**	**0.92**	0.87
	DUD_diverse	**0.96**	0.88	0.84	**0.94**	**0.91**	0.85	**0.93**	0.86	0.79
Ibk	ZINC_random	**0.98**	0.85	0.83	**0.98**	0.82	0.81	**0.97**	0.87	0.84
	ZINC_diverse	**0.99**	**0.94**	**0.93**	**0.99**	0.79	0.76	**0.99**	0.82	0.8
	MDDR_random	**0.97**	0.76	0.73	0.9	0.83	0.74	**0.94**	0.82	0.76
	MDDR_diverse	**0.94**	0.78	0.72	**0.97**	0.79	0.77	**0.98**	0.76	0.76
	DUD_random	**0.96**	**0.96**	**0.92**	**0.98**	0.9	0.87	**0.98**	0.89	0.87
	DUD_diverse	**0.96**	0.89	0.85	**0.98**	0.86	0.84	**0.98**	0.82	0.78
Decorate	ZINC_random	0.77	0.85	0.62	0.81	**0.96**	0.78	0.81	0.84	0.64
	ZINC_diverse	0.74	**0.91**	0.66	**0.97**	**0.92**	0.89	**0.95**	**0.93**	0.88
	MDDR_random	0.78	0.77	0.56	0.8	0.87	0.67	0.76	0.79	0.55
	MDDR_diverse	0.62	0.83	0.45	0.89	0.88	0.77	0.85	0.78	0.63
	DUD_random	**0.96**	**0.94**	0.9	**0.93**	**0.94**	0.87	**0.93**	**0.91**	0.85
	DUD_diverse	**0.95**	0.86	0.8	**0.94**	**0.92**	0.86	0.9	0.87	0.77
Hyperpipes	ZINC_random	**0.99**	0.49	0.6	0.86	0.79	0.65	**0.99**	0.49	0.55
	ZINC_diverse	**0.99**	0.81	0.8	**0.91**	0.78	0.68	**0.99**	0.79	0.77
	MDDR_random	**0.98**	0.52	0.56	0.84	0.81	0.65	**0.98**	0.57	0.6
	MDDR_diverse	**0.98**	0.57	0.6	0.87	0.72	0.59	**0.98**	0.64	0.66
	DUD_random	**0.99**	0.58	0.43	0.89	0.73	0.59	**0.99**	0.59	0.44
	DUD_diverse	**0.98**	0.58	0.42	**0.92**	0.71	0.58	**0.99**	0.6	0.46
J48	ZINC_random	0.85	0.86	0.71	0.88	**0.92**	0.8	0.88	**0.91**	0.79
	ZINC_diverse	**0.94**	0.81	0.75	**0.96**	0.9	0.87	**0.93**	0.88	0.82
	MDDR_random	0.86	0.83	0.69	0.87	0.85	0.72	0.85	0.84	0.69
	MDDR_diverse	0.89	0.74	0.63	**0.91**	0.86	0.76	0.78	0.79	0.57
	DUD_random	0.89	0.89	0.79	0.9	**0.92**	0.83	0.9	0.89	0.79
	DUD_diverse	0.9	0.81	0.7	0.9	0.9	0.8	0.86	0.85	0.72
Random Forest	ZINC_random	**0.93**	**0.97**	0.63	**0.94**	**0.96**	0.9	**0.92**	**0.96**	0.88
	ZINC_diverse	**0.97**	**1**	**0.97**	**0.98**	**0.93**	**0.91**	**0.99**	**0.96**	**0.95**
	MDDR_random	**0.91**	**0.95**	0.86	**0.94**	0.9	0.84	0.89	0.9	0.79
	MDDR_diverse	**0.91**	**0.93**	0.84	**0.95**	0.9	0.85	**0.93**	0.87	0.8
	DUD_random	**0.97**	**0.97**	**0.94**	**0.96**	**0.95**	**0.91**	**0.95**	**0.94**	0.89
	DUD_diverse	**0.96**	**0.91**	0.88	**0.96**	**0.93**	0.9	**0.94**	0.9	0.84

HIV PR (various-test sets mode)										
**ML method**	**inactives selection**	**ExtFP**			**KlekFP**			**MACCSFP**		
		**recall**	**precision**	**MCC**	**recall**	**precision**	**MCC**	**recall**	**precision**	**MCC**
Naïve Bayes	ZINC_random	**0.91**	**0.97**	0.89	**0.93**	**0.92**	0.86	0.85	0.87	0.75
	ZINC_diverse	**0.98**	0.75	0.72	**0.96**	**0.93**	0.89	**0.96**	0.89	0.85
	MDDR_random	0.85	0.87	0.74	0.86	0.84	0.72	0.76	0.82	0.63
	MDDR_diverse	**0.92**	0.84	0.76	**0.92**	0.86	0.79	0.89	0.86	0.76
	DUD_random	**0.99**	**1**	**0.99**	**0.94**	**0.93**	0.88	**0.98**	**0.97**	**0.96**
	DUD_diverse	**0.99**	**0.99**	**0.98**	**0.92**	**0.91**	0.84	**0.95**	**0.94**	0.9
SMO	ZINC_random	**0.99**	**0.97**	**0.97**	**0.98**	**0.97**	**0.96**	**0.95**	**0.94**	0.9
	ZINC_diverse	**1**	0.64	0.58	**0.99**	**0.97**	**0.96**	**0.99**	0.86	0.86
	MDDR_random	**0.95**	**0.93**	0.88	**0.96**	**0.92**	0.88	0.89	0.87	0.78
	MDDR_diverse	**0.97**	**0.95**	**0.93**	**0.97**	**0.93**	0.9	**0.95**	0.9	0.86
	DUD_random	**1**	**1**	**1**	**0.98**	**0.98**	**0.97**	**0.99**	**0.98**	**0.98**
	DUD_diverse	**1**	**1**	**1**	**0.97**	**0.98**	**0.95**	**0.99**	**0.98**	**0.97**
Ibk	ZINC_random	**0.99**	**0.96**	**0.95**	**0.98**	**0.94**	**0.92**	**0.97**	0.87	0.85
	ZINC_diverse	**1**	0.71	0.68	**0.99**	**0.94**	**0.94**	**1**	0.83	0.83
	MDDR_random	**0.98**	0.87	0.85	**0.97**	0.88	0.86	**0.96**	0.79	0.75
	MDDR_diverse	**0.99**	0.89	0.88	**0.98**	**0.93**	**0.91**	**0.98**	0.81	0.79
	DUD_random	**1**	**1**	**1**	**0.98**	**0.97**	**0.96**	**0.99**	**0.98**	**0.98**
	DUD_diverse	**1**	**1**	**1**	**0.98**	**0.96**	**0.94**	**0.99**	**0.96**	**0.95**
Decorate	ZINC_random	**0.95**	**0.97**	**0.92**	**0.95**	**0.96**	**0.92**	**0.91**	**0.94**	0.87
	ZINC_diverse	**1**	0.49	0.25	**0.98**	**0.98**	**0.96**	**0.99**	0.87	0.86
	MDDR_random	**0.91**	**0.93**	0.85	**0.93**	**0.93**	0.86	0.88	0.89	0.79
	MDDR_diverse	**0.97**	**0.94**	**0.91**	**0.96**	**0.92**	0.89	**0.93**	**0.91**	0.85
	DUD_random	**1**	**1**	**1**	**0.96**	**0.97**	**0.94**	**0.99**	**0.98**	**0.98**
	DUD_diverse	**0.99**	**1**	**1**	**0.96**	**0.97**	**0.94**	**1**	**0.96**	**0.96**
Hyperpipes	ZINC_random	**1**	0.46	0.1	**0.91**	0.74	0.64	**0.99**	0.51	0.29
	ZINC_diverse	**1**	0.51	0.32	**0.95**	0.87	0.82	**0.98**	0.79	0.77
	MDDR_random	**1**	0.47	0.14	0.86	0.76	0.63	**0.98**	0.55	0.38
	MDDR_diverse	**1**	0.52	0.33	0.89	0.78	0.68	**0.98**	0.63	0.55
	DUD_random	**1**	**1**	**1**	**0.96**	0.79	0.74	**1**	**0.98**	**0.98**
	DUD_diverse	**1**	**1**	**1**	**0.94**	0.76	0.69	**0.99**	**0.94**	**0.94**
J48	ZINC_random	**0.93**	0.9	0.84	**0.94**	**0.94**	0.88	0.88	0.9	0.8
	ZINC_diverse	**1**	0.53	0.37	**0.95**	**0.97**	**0.93**	**0.98**	0.87	0.85
	MDDR_random	0.86	0.84	0.72	0.9	0.9	0.82	0.84	0.84	0.71
	MDDR_diverse	**0.92**	0.85	0.77	**0.94**	0.9	0.85	**0.95**	0.83	0.79
	DUD_random	**0.99**	**0.99**	**0.99**	**0.94**	**0.96**	**0.91**	**0.99**	**0.98**	**0.98**
	DUD_diverse	**0.99**	**0.99**	**0.98**	**0.94**	**0.96**	**0.92**	**0.98**	**0.97**	**0.95**
Random Forest	ZINC_random	**0.95**	**0.99**	**0.95**	**0.97**	**0.99**	**0.96**	**0.95**	**0.97**	**0.93**
	ZINC_diverse	**1**	0.77	0.76	**0.98**	**0.98**	**0.96**	**1**	**0.92**	**0.92**
	MDDR_random	0.9	**0.98**	0.89	**0.95**	**0.96**	**0.92**	**0.92**	**0.93**	0.86
	MDDR_diverse	**0.97**	**0.98**	**0.95**	**0.96**	**0.96**	**0.93**	**0.97**	**0.94**	**0.91**
	DUD_random	**1**	**1**	**1**	**0.97**	**0.98**	**0.96**	**1**	**0.99**	**0.98**
	DUD_diverse	**1**	**1**	**1**	**0.96**	**0.99**	**0.96**	**0.99**	**0.97**	**0.97**

metalloproteinase (various-test sets mode)										
**ML method**	**inactives selection**	**ExtFP**			**KlekFP**			**MACCSFP**		
		**recall**	**precision**	**MCC**	**recall**	**precision**	**MCC**	**recall**	**precision**	**MCC**
Naïve Bayes	ZINC_random	0.82	**0.92**	0.78	0.87	0.88	0.77	0.76	0.81	0.62
	ZINC_diverse	**0.97**	0.66	0.6	**0.95**	**0.91**	0.87	**0.95**	0.9	0.86
	MDDR_random	0.84	0.73	0.58	0.87	0.86	0.75	0.74	0.81	0.61
	MDDR_diverse	0.89	**0.93**	0.84	**0.92**	0.86	0.79	0.88	0.85	0.75
	DUD_random	**1**	**0.99**	**0.99**	0.88	0.87	0.77	**0.99**	**0.95**	**0.95**
	DUD_diverse	**0.99**	**1**	**0.98**	0.89	0.88	0.79	**0.96**	0.86	0.83
SMO	ZINC_random	**0.91**	**0.96**	0.89	**0.94**	**0.96**	**0.92**	**0.91**	**0.92**	0.85
	ZINC_diverse	**1**	0.58	0.49	**0.97**	**0.97**	**0.95**	**0.98**	**0.93**	**0.92**
	MDDR_random	0.88	**0.92**	0.82	0.9	**0.92**	0.84	0.83	0.87	0.73
	MDDR_diverse	**0.93**	**0.95**	0.89	**0.95**	**0.94**	0.9	**0.93**	0.9	0.84
	DUD_random	**1**	**1**	**1**	**0.95**	**0.96**	**0.92**	**0.99**	**0.97**	**0.96**
	DUD_diverse	**1**	**1**	**1**	**0.95**	**0.95**	**0.91**	**0.98**	**0.91**	0.9
Ibk	ZINC_random	**0.93**	0.9	0.85	**0.93**	**0.91**	0.85	**0.94**	0.88	0.83
	ZINC_diverse	**0.99**	0.69	0.65	**0.97**	**0.94**	**0.92**	**0.99**	0.85	0.85
	MDDR_random	**0.94**	0.79	0.74	**0.93**	0.87	0.82	**0.94**	0.81	0.76
	MDDR_diverse	**0.95**	0.85	0.81	**0.94**	**0.92**	0.87	**0.97**	0.81	0.79
	DUD_random	**1**	**1**	**1**	**0.93**	**0.94**	0.89	**0.98**	**0.97**	**0.95**
	DUD_diverse	**0.99**	**1**	**0.99**	**0.94**	**0.91**	0.87	**0.99**	**0.92**	**0.91**
Decorate	ZINC_random	0.87	**0.94**	0.84	0.9	**0.96**	0.87	0.88	**0.94**	0.84
	ZINC_diverse	**1**	0.49	0.27	**0.95**	**0.96**	**0.93**	**0.96**	0.9	0.87
	MDDR_random	0.81	**0.92**	0.77	0.87	**0.94**	0.84	0.86	0.89	0.77
	MDDR_diverse	**0.91**	**0.92**	0.85	**0.92**	**0.96**	0.89	**0.94**	**0.91**	0.86
	DUD_random	**0.94**	**1**	**0.95**	**0.91**	**0.96**	0.88	**0.99**	**0.96**	**0.96**
	DUD_diverse	**1**	**1**	**1**	**0.92**	**0.97**	**0.91**	**1**	0.89	0.89
Hyperpipes	ZINC_random	**1**	0.45	0.04	**0.93**	0.71	0.62	**0.99**	0.49	0.28
	ZINC_diverse	**0.99**	0.49	0.28	**0.98**	0.87	0.86	**0.98**	0.77	0.74
	MDDR_random	**0.99**	0.46	0.16	0.84	0.72	0.58	**0.98**	0.56	0.43
	MDDR_diverse	**0.99**	0.51	0.32	0.88	0.73	0.62	**0.98**	0.62	0.54
	DUD_random	**1**	**1**	**0.99**	**0.96**	0.73	0.68	**1**	0.47	0.22
	DUD_diverse	**0.99**	**0.98**	**0.98**	**0.95**	0.74	0.69	**0.99**	0.51	0.32
J48	ZINC_random	0.82	0.83	0.68	0.87	**0.93**	0.83	0.84	0.89	0.76
	ZINC_diverse	**0.99**	0.65	0.59	**0.94**	**0.96**	**0.91**	**0.94**	0.89	0.84
	MDDR_random	0.76	0.77	0.58	0.83	**0.91**	0.77	0.83	0.84	0.7
	MDDR_diverse	0.87	0.86	0.76	**0.92**	**0.91**	0.85	0.89	0.87	0.78
	DUD_random	**1**	**0.99**	**0.99**	0.88	**0.94**	0.85	**0.97**	**0.96**	**0.93**
	DUD_diverse	**0.99**	**0.99**	**0.98**	0.9	**0.93**	0.84	**0.97**	0.87	0.85
Random Forest	ZINC_random	0.87	**0.99**	0.88	0.9	**0.98**	0.89	0.9	**0.96**	0.87
	ZINC_diverse	**0.99**	0.7	0.67	**0.96**	**0.96**	**0.93**	**0.98**	**0.93**	**0.91**
	MDDR_random	0.82	**0.97**	0.82	0.9	**0.95**	0.87	0.88	**0.92**	0.82
	MDDR_diverse	**0.91**	**0.98**	**0.91**	**0.96**	**0.93**	0.9	**0.95**	**0.93**	0.89
	DUD_random	**1**	**1**	**1**	**0.92**	**0.98**	**0.91**	**1**	**0.97**	**0.96**
	DUD_diverse	**1**	**1**	**1**	**0.93**	**0.98**	**0.92**	**1**	**0.91**	**0.91**

5-HT1A (various-test sets mode)										
**ML method**	**inactives selection**	**ExtFP**			**KlekFP**			**MACCSFP**		
		**recall**	**precision**	**MCC**	**recall**	**precision**	**MCC**	**recall**	**precision**	**MCC**
Naïve Bayes	ZINC_random	0.86	0.86	0.74	0.88	**0.91**	0.8	0.87	0.8	0.68
	ZINC_diverse	**0.99**	0.65	0.59	**0.95**	0.89	0.85	**0.92**	0.84	0.76
	MDDR_random	0.88	0.7	0.57	0.89	0.81	0.71	0.83	0.72	0.55
	MDDR_diverse	**0.94**	0.86	0.81	**0.93**	0.84	0.77	**0.92**	0.72	0.62
	DUD_random	**1**	**0.99**	**0.99**	**0.91**	0.85	0.77	**0.95**	**0.97**	**0.93**
	DUD_diverse	**1**	**0.98**	**0.98**	**0.91**	0.82	0.73	**0.95**	**0.92**	0.88
SMO	ZINC_random	**0.95**	**0.95**	**0.91**	**0.94**	**0.94**	0.89	**0.93**	**0.93**	0.87
	ZINC_diverse	1	0.58	0.48	**0.96**	**0.95**	**0.92**	**0.99**	0.84	0.83
	MDDR_random	**0.92**	0.9	0.83	**0.92**	0.88	0.81	0.84	0.84	0.71
	MDDR_diverse	**0.95**	**0.93**	0.89	**0.94**	0.9	0.85	**0.92**	0.83	0.76
	DUD_random	**1**	**1**	**1**	**0.94**	**0.94**	0.89	**1**	**0.99**	**0.99**
	DUD_diverse	**1**	**1**	**1**	**0.94**	**0.92**	0.87	**0.99**	**0.95**	**0.94**
Ibk	ZINC_random	**0.96**	0.88	0.84	**0.95**	0.84	0.8	**0.96**	0.85	0.81
	ZINC_diverse	**1**	0.69	0.65	**0.97**	0.9	0.87	**1**	0.75	0.73
	MDDR_random	**0.97**	0.77	0.73	**0.94**	0.79	0.73	**0.94**	0.76	0.69
	MDDR_diverse	**0.97**	0.8	0.76	**0.94**	0.86	0.81	**0.97**	0.75	0.71
	DUD_random	**1**	**1**	**1**	**0.95**	**0.91**	0.86	**0.98**	**0.98**	**0.97**
	DUD_diverse	**1**	**1**	**1**	**0.94**	0.87	0.81	**0.99**	**0.92**	**0.92**
Decorate	ZINC_random	0.87	**0.91**	0.81	0.9	**0.93**	0.84	**0.92**	**0.94**	0.86
	ZINC_diverse	**1**	0.5	0.25	**0.96**	**0.96**	**0.92**	**0.99**	0.83	0.82
	MDDR_random	0.86	0.87	0.75	0.89	0.88	0.78	0.85	0.85	0.72
	MDDR_diverse	**0.93**	0.87	0.81	**0.94**	**0.91**	0.85	0.88	0.82	0.71
	DUD_random	**1**	**1**	**1**	**0.93**	**0.93**	0.87	**1**	**0.99**	**0.99**
	DUD_diverse	**1**	**1**	**1**	0.9	**0.92**	0.84	**1**	**0.91**	**0.92**
Hyperpipes	ZINC_random	**1**	0.5	0.25	0.87	0.81	0.69	**0.99**	0.52	0.31
	ZINC_diverse	**1**	0.59	0.49	**0.91**	0.84	0.76	**0.99**	0.82	0.81
	MDDR_random	**1**	0.52	0.32	0.84	0.82	0.68	**0.99**	0.56	0.42
	MDDR_diverse	**1**	0.6	0.51	0.87	0.79	0.67	**0.99**	0.64	0.57
	DUD_random	**1**	**1**	**1**	0.9	0.8	0.71	**1**	**0.96**	**0.96**
	DUD_diverse	**1**	**1**	**1**	0.9	0.79	0.69	**1**	0.89	0.89
J48	ZINC_random	0.81	0.83	0.67	0.87	0.9	0.78	0.88	**0.91**	0.81
	ZINC_diverse	**0.99**	0.47	0.1	**0.92**	**0.92**	0.86	**0.99**	0.82	0.8
	MDDR_random	0.77	0.77	0.57	0.86	0.85	0.73	0.82	0.81	0.65
	MDDR_diverse	0.85	0.8	0.67	0.9	0.83	0.74	0.89	0.81	0.71
	DUD_random	**0.99**	**0.99**	**0.99**	**0.91**	0.9	0.82	**0.99**	**0.99**	**0.98**
	DUD_diverse	**1**	**0.99**	**0.98**	**0.94**	**0.92**	0.87	**1**	**0.91**	**0.91**
Random Forest	ZINC_random	0.88	**0.98**	0.88	**0.92**	**0.96**	0.89	**0.94**	**0.95**	0.9
	ZINC_diverse	**1**	0.65	0.59	**0.97**	**0.95**	**0.92**	**0.99**	0.87	0.86
	MDDR_random	0.86	**0.95**	0.83	**0.91**	**0.91**	0.83	0.88	0.9	0.8
	MDDR_diverse	**0.94**	**0.95**	0.9	**0.95**	**0.94**	0.9	**0.94**	0.88	0.83
	DUD_random	**1**	**1**	**1**	**0.94**	**0.95**	0.9	**0.99**	**0.99**	**0.99**
	DUD_diverse	**1**	**1**	**1**	**0.94**	**0.94**	0.89	**0.99**	**0.95**	**0.94**

Table S3. Standard deviation of evaluating parameters values.

**Standard deviation (common-test set mode)**										
**ML method**	**inactives selection**	**ExtFP**			**KlekFP**			**MACCSFP**		
		**recall**	**precision**	**MCC**	**recall**	**precision**	**MCC**	**recall**	**precision**	**MCC**
COX-2	Naïve Bayes	**0.03**	0.187	0.23	**0.006**	**0.029**	**0.032**	**0.013**	**0.05**	**0.047**
	SMO	**0**	0.255	0.376	**0.003**	**0**	**0.003**	**0.007**	**0.058**	**0.054**
	Ibk	**0.006**	0.122	0.133	**0.015**	**0.007**	**0.016**	**0.012**	**0.041**	**0.039**
	Decorate	**0.002**	0.212	0.277	**0.005**	**0**	**0.005**	**0.009**	0.16	0.2
	Hyperpipes	**0.001**	**0.053**	**0.055**	**0**	**0.049**	**0.06**	**0.004**	**0.019**	**0.038**
	J48	**0.011**	0.182	0.256	**0.006**	**0.005**	**0.009**	**0.01**	0.158	0.2
	Random Forest	**0.007**	0.168	0.203	**0.007**	**0.008**	**0.011**	**0.007**	0.107	0.119
M_1	Naïve Bayes	0.121	0.137	0.135	**0.039**	**0.097**	**0.099**	**0.05**	**0.064**	**0.073**
	SMO	**0.037**	0.184	0.298	**0.014**	0.132	0.167	**0.019**	0.139	0.219
	Ibk	**0.016**	0.173	0.212	**0.02**	0.101	0.129	**0.02**	0.12	0.179
	Decorate	0.123	0.125	0.221	**0.025**	0.11	0.124	**0.026**	0.139	0.206
	Hyperpipes	**0.003**	**0.05**	0.233	**0.028**	**0.039**	**0.056**	**0.004**	**0.003**	**0.064**
	J48	**0.052**	0.144	0.203	**0.031**	0.111	0.125	**0.036**	0.13	0.232
	Random Forest	**0.031**	0.18	0.284	**0.025**	0.106	0.111	**0.03**	0.135	0.175
HIV PR	Naïve Bayes	**0.055**	0.215	0.281	**0.036**	0.108	0.107	**0.083**	0.131	0.14
	SMO	**0.019**	0.235	0.377	**0.014**	**0.086**	0.102	**0.04**	0.158	0.2
	Ibk	**0.006**	0.179	0.239	**0.006**	**0.055**	**0.057**	**0.016**	0.109	0.138
	Decorate	**0.035**	0.229	0.32	**0.017**	**0.077**	**0.09**	**0.05**	0.178	0.222
	Hyperpipes	**0.005**	**0.018**	**0.05**	**0.084**	**0.062**	**0.079**	**0.007**	**0.007**	**0.029**
	J48	**0.053**	0.178	0.279	**0.019**	**0.07**	**0.089**	**0.063**	0.154	0.203
	Random Forest	**0.038**	0.233	0.3	**0.012**	**0.075**	**0.084**	**0.033**	0.187	0.231
metalloproteinase	Naïve Bayes	**0.08**	0.235	0.343	**0.03**	0.126	0.146	0.101	0.108	**0.071**
	SMO	**0.045**	0.265	0.438	**0.011**	0.118	0.125	**0.036**	0.159	0.169
	Ibk	**0.028**	0.209	0.324	**0.015**	**0.091**	**0.094**	**0.024**	0.13	0.152
	Decorate	**0.06**	0.24	0.392	**0.023**	**0.076**	**0.064**	**0.052**	0.189	0.218
	Hyperpipes	**0.002**	**0.003**	**0.02**	**0.035**	**0.046**	**0.071**	**0.008**	**0.003**	**0.012**
	J48	**0.087**	0.171	0.245	**0.026**	**0.082**	**0.068**	**0.057**	0.164	0.189
	Random Forest	**0.061**	0.276	0.407	**0.026**	0.115	0.104	**0.045**	0.18	0.186
$5\text{-}HT_{1A}$	Naïve Bayes	**0.062**	0.156	0.26	**0.027**	0.126	0.15	**0.047**	**0.092**	0.112
	SMO	**0.035**	0.235	0.407	**0.015**	0.1	0.115	**0.062**	0.139	0.157
	Ibk	**0.019**	0.148	0.237	**0.011**	**0.061**	**0.077**	**0.022**	**0.099**	0.127
	Decorate	**0.067**	0.214	0.352	**0.028**	**0.09**	**0.09**	**0.067**	0.171	0.209
	Hyperpipes	**0.001**	**0.008**	**0.033**	**0.028**	**0.049**	**0.047**	**0.001**	**0.002**	**0.004**
	J48	0.101	0.165	0.266	**0.031**	**0.097**	0.105	**0.075**	0.153	0.192
	Random Forest	**0.064**	0.248	0.371	**0.02**	**0.096**	**0.095**	**0.045**	0.159	0.178

Standard deviation (various-test sets mode)										
**ML method**	**inactives selection**	**ExtFP**			**KlekFP**			**MACCSFP**		
		**recall**	**precision**	**MCC**	**recall**	**precision**	**MCC**	**recall**	**precision**	**MCC**
COX-2	Naïve Bayes	**0.029**	**0.082**	**0.094**	**0.006**	**0.028**	**0.027**	**0.013**	**0.032**	**0.041**
	SMO	**0**	0.123	0.144	**0.003**	**0**	**0.004**	**0.008**	**0.02**	**0.022**
	Ibk	**0.004**	**0.087**	**0.096**	**0.013**	**0.003**	**0.022**	**0.011**	**0.033**	**0.035**
	Decorate	**0.003**	0.119	0.139	**0.005**	**0**	**0.006**	**0.009**	**0.005**	**0.009**
	Hyperpipes	**0.001**	**0.05**	**0.051**	**0**	**0.059**	**0.085**	**0.004**	0.162	0.199
	J48	**0.006**	**0.083**	**0.089**	**0.006**	**0.006**	**0.008**	**0.009**	**0.013**	**0.02**
	Random Forest	**0.003**	**0.074**	**0.078**	**0.006**	**0.003**	**0.009**	**0.007**	**0.013**	**0.016**
M_1	Naïve Bayes	0.105	**0.053**	0.138	**0.052**	**0.017**	**0.058**	**0.05**	**0.086**	0.103
	SMO	**0.03**	**0.041**	**0.054**	**0.025**	**0.023**	**0.029**	**0.042**	**0.042**	**0.068**
	Ibk	**0.018**	**0.085**	**0.091**	**0.032**	**0.041**	**0.05**	**0.016**	**0.046**	**0.045**
	Decorate	0.131	**0.059**	0.163	**0.071**	**0.032**	**0.082**	**0.072**	**0.064**	0.132
	Hyperpipes	**0.002**	0.114	0.139	**0.029**	**0.043**	**0.04**	**0.002**	0.102	0.125
	J48	**0.032**	**0.051**	**0.055**	**0.033**	**0.031**	**0.052**	**0.05**	**0.045**	**0.093**
	Random Forest	**0.029**	**0.031**	0.121	**0.015**	**0.023**	**0.031**	**0.032**	**0.036**	**0.061**
HIV PR	Naïve Bayes	**0.058**	0.1	0.123	**0.034**	**0.038**	**0.066**	**0.082**	**0.056**	0.12
	SMO	**0.02**	0.138	0.16	**0.013**	**0.026**	**0.035**	**0.04**	**0.052**	**0.076**
	Ibk	**0.006**	0.11	0.12	**0.006**	**0.033**	**0.036**	**0.015**	**0.079**	**0.091**
	Decorate	**0.036**	0.196	0.284	**0.017**	**0.025**	**0.036**	**0.05**	**0.045**	**0.073**
	Hyperpipes	**0.001**	0.263	0.412	**0.038**	**0.044**	**0.072**	**0.007**	0.202	0.289
	J48	**0.054**	0.169	0.226	**0.019**	**0.031**	**0.043**	**0.063**	**0.063**	0.104
	Random Forest	**0.039**	**0.089**	**0.09**	**0.01**	**0.013**	**0.019**	**0.033**	**0.027**	**0.045**
metalloproteinase	Naïve Bayes	**0.077**	0.142	0.179	**0.031**	**0.021**	**0.041**	0.105	**0.056**	0.135
	SMO	**0.052**	0.161	0.189	**0.023**	**0.02**	**0.038**	**0.061**	**0.035**	**0.081**
	Ibk	**0.029**	0.122	0.138	**0.016**	**0.026**	**0.035**	**0.025**	**0.063**	**0.075**
	Decorate	**0.074**	0.195	0.265	**0.028**	**0.009**	**0.031**	**0.058**	**0.032**	**0.06**
	Hyperpipes	**0.002**	0.266	0.418	**0.053**	**0.061**	**0.099**	**0.008**	0.111	0.194
	J48	0.103	0.131	0.184	**0.036**	**0.02**	**0.044**	**0.062**	**0.041**	**0.081**
	Random Forest	**0.077**	0.119	0.125	**0.027**	**0.018**	**0.021**	**0.052**	**0.023**	**0.048**
5-HT_{1A}	Naïve Bayes	**0.062**	0.141	0.184	**0.027**	**0.039**	**0.049**	**0.047**	0.104	0.148
	SMO	**0.035**	0.157	0.195	**0.014**	**0.029**	**0.04**	**0.062**	**0.068**	0.108
	Ibk	**0.019**	0.127	0.146	**0.011**	**0.043**	**0.052**	**0.022**	**0.098**	0.115
	Decorate	**0.067**	0.187	0.273	**0.027**	**0.025**	**0.045**	**0.067**	**0.067**	0.109
	Hyperpipes	**0.001**	0.235	0.331	**0.028**	**0.02**	**0.034**	**0.003**	0.184	0.265
	J48	0.101	0.191	0.327	**0.031**	**0.04**	**0.06**	**0.075**	**0.074**	0.122
	Random Forest	**0.064**	0.136	0.152	**0.02**	**0.019**	**0.031**	**0.045**	**0.049**	**0.071**
