# Supplementary figures and images for: The influence of the inactives subset generation on the performance of machine learning methods
Source: J Cheminform. 2013 Apr 5;5:17. doi: 10.1186/1758-2946-5-17 (PMC3626618; doi:10.1186/1758-2946-5-17)

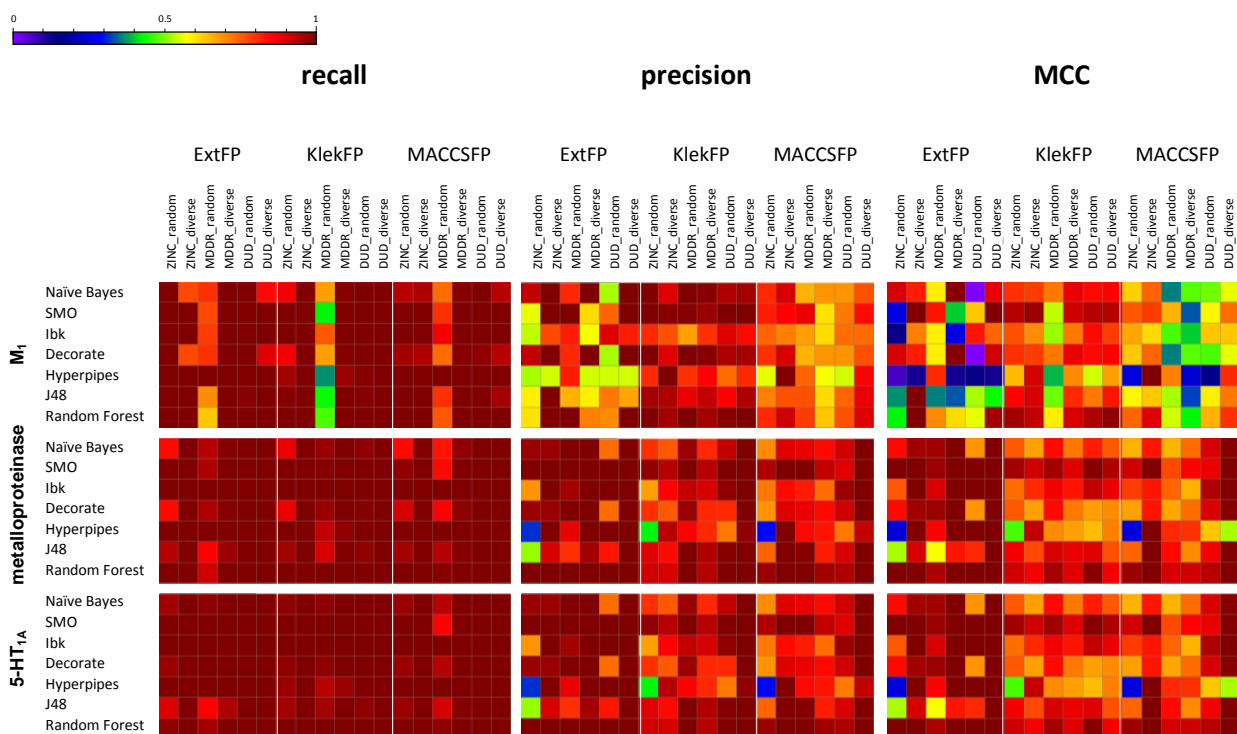

Figure S2. Evaluating parameters value obtained for sets with inactives selected from PubChem.

Supplement: Additional file 3: Figure S2 — A a panel of heat maps obtained in tests with external sets of in actives fetched from PubChem database. [file 1758-2946-5-17-S3.pdf]
