# Supplementary material for: The influence of the inactives subset generation on the performance of machine learning methods
Source: J Cheminform. 2013 Apr 5;5:17. doi: 10.1186/1758-2946-5-17 (PMC3626618; doi:10.1186/1758-2946-5-17)
Supplement: Additional file 4: Tables S4 — Numerical values of evaluating parameters obtained in experiments with inactives from PubChem database. [file 1758-2946-5-17-S4.pdf]

Table S4. Evaluating parameters values obtained in experiments with inactives from PubChem database

M_1										
**ML method**	**inactives selection**	**ExtFP**			**KlekFP**			**MACCSFP**		
		**recall**	**precision**	**MCC**	**recall**	**precision**	**MCC**	**recall**	**precision**	**MCC**
Naïve Bayes	ZINC_random	**0.99**	0.83	0.81	0.78	**0.91**	0.72	0.84	0.73	0.56
	ZINC_diverse	0.70	**1.00**	0.74	**0.91**	0.80	0.71	0.85	0.81	0.67
	MDDR_random	0.72	0.73	0.53	0.61	**0.90**	0.65	0.66	0.59	0.32
	MDDR_diverse	**0.96**	**0.93**	0.89	0.89	0.89	0.79	0.89	0.62	0.43
	DUD_random	**0.93**	0.47	-0.04	**0.90**	0.85	0.76	0.90	0.62	0.44
	DUD_diverse	0.76	**1.00**	0.79	**0.91**	0.86	0.77	0.84	0.69	0.50
SMO	ZINC_random	**1.00**	0.50	0.23	**0.90**	**0.92**	0.84	**0.97**	0.74	0.68
	ZINC_diverse	**0.98**	**1.00**	**0.98**	**0.98**	0.89	0.87	**0.96**	0.77	0.71
	MDDR_random	0.70	**0.99**	0.75	0.38	0.89	0.48	0.72	0.79	0.59
	MDDR_diverse	**1.00**	0.55	0.36	**0.94**	0.87	0.82	**0.97**	0.54	0.30
	DUD_random	**0.98**	0.67	0.57	**0.93**	**0.91**	0.84	**0.94**	0.65	0.52
	DUD_diverse	**0.95**	**0.96**	**0.91**	**0.91**	**0.92**	0.84	**0.92**	0.76	0.66
Ibk	ZINC_random	**1.00**	0.48	0.12	**0.98**	0.73	0.69	**0.99**	0.67	0.60
	ZINC_diverse	**0.99**	0.70	0.64	**0.99**	0.69	0.63	**0.99**	0.64	0.55
	MDDR_random	0.71	0.74	0.53	0.68	0.61	0.46	0.78	0.61	0.42
	MDDR_diverse	**1.00**	0.51	0.23	**0.97**	0.72	0.65	**0.99**	0.55	0.36
	DUD_random	**0.96**	0.80	0.75	**0.98**	0.80	0.76	**0.98**	0.66	0.57
	DUD_diverse	**0.96**	0.74	0.67	**0.98**	0.76	0.71	**0.98**	0.66	0.57
Decorate	ZINC_random	**0.99**	0.83	0.81	0.78	**0.91**	0.72	0.86	0.72	0.57
	ZINC_diverse	0.70	**1.00**	0.74	**0.91**	0.80	0.71	0.85	0.81	0.67
	MDDR_random	0.72	0.73	0.53	0.61	**0.90**	0.65	0.66	0.59	0.32
	MDDR_diverse	**0.96**	**0.93**	0.89	0.89	0.89	0.79	0.89	0.62	0.43
	DUD_random	**0.93**	0.47	-0.04	**0.90**	0.85	0.76	0.88	0.62	0.42
	DUD_diverse	0.80	**1.00**	0.82	**0.91**	0.86	0.77	0.84	0.69	0.50
Hyperpipes	ZINC_random	**0.99**	0.47	0.06	0.86	0.73	0.58	**0.99**	0.50	0.22
	ZINC_diverse	**0.98**	0.49	0.11	**0.91**	0.90	0.81	**0.99**	**0.98**	**0.97**
	MDDR_random	**0.99**	0.74	0.73	0.33	0.72	0.35	**0.99**	0.67	0.64
	MDDR_diverse	**0.98**	0.49	0.11	0.87	0.77	0.63	**0.98**	0.51	0.21
	DUD_random	**0.99**	0.49	0.12	0.89	0.66	0.49	**0.99**	0.49	0.14
	DUD_diverse	**0.98**	0.49	0.11	**0.92**	0.73	0.61	**0.99**	0.77	0.73
J48	ZINC_random	**1.00**	0.53	0.33	0.89	0.86	0.77	**0.95**	0.64	0.52
	ZINC_diverse	**0.92**	**1.00**	**0.93**	**0.96**	0.85	0.81	**0.93**	0.69	0.57
	MDDR_random	0.63	0.59	0.32	0.39	0.79	0.44	0.72	0.69	0.47
	MDDR_diverse	**0.99**	0.53	0.30	**0.90**	0.83	0.73	**0.94**	0.55	0.29
	DUD_random	0.89	0.65	0.47	0.89	0.77	0.65	0.90	0.67	0.53
	DUD_diverse	0.90	0.60	0.39	0.90	0.85	0.75	0.86	0.79	0.65
Random Forest	ZINC_random	**1.00**	0.54	0.38	**0.94**	**0.91**	0.86	**0.98**	0.73	0.67
	ZINC_diverse	**0.96**	**1.00**	**0.97**	**0.98**	0.86	0.83	**0.99**	0.82	0.80
	MDDR_random	0.57	**0.95**	0.63	0.42	**0.92**	0.53	0.68	0.71	0.49
	MDDR_diverse	**1.00**	0.64	0.55	**0.96**	0.86	0.81	**0.96**	0.57	0.38
	DUD_random	**0.97**	0.63	0.50	**0.96**	0.89	0.85	**0.95**	0.69	0.59
	DUD_diverse	**0.96**	0.90	0.86	**0.96**	**0.91**	0.88	**0.94**	0.80	0.72

metalloproteinase										
**ML method**	**inactives selection**	**ExtFP**			**KlekFP**			**MACCSFP**		
		**recall**	**precision**	**MCC**	**recall**	**precision**	**MCC**	**recall**	**precision**	**MCC**
Naïve Bayes	ZINC_random	0.76	0.87	0.76	0.79	0.72	0.68	0.76	0.63	0.59
	ZINC_diverse	**0.97**	0.87	0.86	**0.95**	0.68	0.61	**0.95**	0.80	0.75
	MDDR_random	0.84	**1.00**	0.86	0.87	0.87	0.76	0.75	0.79	0.59
	MDDR_diverse	0.89	**1.00**	**0.91**	**0.92**	0.73	0.65	0.88	0.76	0.66
	DUD_random	**1.00**	0.66	0.62	0.88	0.83	0.74	**0.99**	0.82	0.81
	DUD_diverse	**0.99**	**1.00**	**0.98**	0.90	**0.95**	0.68	**0.97**	**0.98**	0.89
SMO	ZINC_random	**0.90**	**0.95**	**0.90**	**0.90**	0.88	0.86	0.88	0.85	0.82
	ZINC_diverse	**1.00**	**1.00**	**1.00**	**0.96**	0.84	0.82	**0.97**	**0.91**	0.90
	MDDR_random	0.85	**1.00**	0.87	0.89	**0.96**	0.86	0.76	0.90	0.70
	MDDR_diverse	**0.91**	**1.00**	**0.93**	**0.94**	0.84	0.80	**0.92**	0.83	0.77
	DUD_random	**1.00**	**0.99**	**0.99**	**0.94**	**0.94**	0.89	**0.99**	0.80	0.79
	DUD_diverse	**0.90**	**0.95**	**0.90**	**0.90**	0.88	0.86	0.88	0.85	0.82
Ibk	ZINC_random	**0.94**	0.62	0.68	**0.92**	0.61	0.66	**0.95**	0.65	0.71
	ZINC_diverse	**0.99**	**0.99**	**0.98**	**0.97**	0.77	0.73	**0.99**	0.76	0.75
	MDDR_random	**0.93**	0.86	0.81	**0.93**	0.83	0.78	**0.93**	0.74	0.67
	MDDR_diverse	**0.95**	**0.95**	**0.91**	**0.94**	0.81	0.75	**0.97**	0.66	0.59
	DUD_random	**0.99**	**0.97**	**0.96**	**0.94**	0.88	0.83	**0.98**	0.87	0.85
	DUD_diverse	**0.99**	**1.00**	**0.99**	**0.96**	**0.95**	0.79	**0.99**	**0.97**	**0.92**
Decorate	ZINC_random	0.76	0.87	0.76	0.79	0.72	0.68	0.81	0.62	0.61
	ZINC_diverse	**0.97**	0.87	0.86	**0.95**	0.68	0.61	**0.95**	0.80	0.75
	MDDR_random	0.85	**1.00**	0.87	0.87	0.87	0.76	0.77	0.79	0.60
	MDDR_diverse	0.89	**1.00**	**0.91**	**0.92**	0.73	0.65	0.88	0.76	0.66
	DUD_random	**1.00**	0.66	0.62	0.89	0.73	0.61	**0.99**	0.82	0.81
	DUD_diverse	**0.98**	**1.00**	**0.94**	**0.91**	**0.93**	0.63	**0.97**	**0.98**	0.89
Hyperpipes	ZINC_random	**1.00**	0.28	0.22	**0.95**	0.38	0.42	**1.00**	0.26	0.22
	ZINC_diverse	**0.99**	**0.95**	**0.95**	**0.98**	0.84	0.83	**0.98**	**0.98**	**0.96**
	MDDR_random	**0.99**	0.79	0.77	0.83	0.77	0.63	**0.98**	0.76	0.73
	MDDR_diverse	**0.99**	**0.95**	**0.95**	0.88	0.73	0.61	**0.98**	0.75	0.72
	DUD_random	**1.00**	**0.95**	**0.95**	**0.97**	0.65	0.58	**0.99**	0.65	0.57
	DUD_diverse	**1.00**	**0.99**	**0.98**	**0.98**	0.88	0.64	**1.00**	0.83	0.47
J48	ZINC_random	0.84	0.46	0.47	0.86	0.80	0.78	0.86	0.67	0.67
	ZINC_diverse	**0.99**	0.81	0.81	**0.94**	0.76	0.70	**0.94**	**0.95**	**0.90**
	MDDR_random	0.77	0.72	0.51	0.81	**0.96**	0.81	0.84	0.89	0.76
	MDDR_diverse	0.87	0.86	0.75	**0.92**	0.85	0.79	0.89	0.74	0.63
	DUD_random	**0.99**	0.75	0.73	0.88	0.89	0.80	**0.96**	0.81	0.78
	DUD_diverse	**0.99**	**0.99**	**0.96**	**0.90**	**0.95**	0.69	**0.97**	**0.98**	**0.90**
Random Forest	ZINC_random	**0.91**	**0.92**	0.90	**0.91**	0.82	0.82	**0.92**	0.87	0.87
	ZINC_diverse	**0.99**	**1.00**	**0.99**	**0.96**	0.81	0.78	**0.98**	**0.93**	**0.92**
	MDDR_random	0.82	**1.00**	0.85	0.90	**0.94**	0.86	0.89	**0.90**	0.81
	MDDR_diverse	**0.91**	**1.00**	**0.92**	**0.96**	0.84	0.80	**0.95**	0.86	0.82
	DUD_random	**1.00**	**0.96**	**0.96**	**0.92**	**0.95**	0.89	**0.99**	0.85	0.84
	DUD_diverse	**1.00**	**1.00**	**1.00**	**0.97**	**0.94**	0.78	**1.00**	**0.99**	**0.96**

5-HT$_{1A}$										
**ML method**	**inactives selection**	**ExtFP**			**KlekFP**			**MACCSFP**		
		**recall**	**precision**	**MCC**	**recall**	**precision**	**MCC**	**recall**	**precision**	**MCC**
Naïve Bayes	ZINC_random	0.86	**1.00**	0.88	0.88	0.87	0.77	0.87	0.86	0.75
	ZINC_diverse	**0.99**	**1.00**	**0.99**	**0.95**	0.65	0.54	**0.92**	0.80	0.72
	MDDR_random	0.88	0.84	0.73	0.88	0.82	0.71	0.84	0.82	0.68
	MDDR_diverse	**0.94**	**1.00**	**0.95**	**0.93**	0.71	0.62	**0.92**	0.80	0.72
	DUD_random	**1.00**	0.88	0.88	**0.91**	0.74	0.64	**0.95**	0.81	0.76
	DUD_diverse	**1.00**	**0.93**	**0.92**	**0.91**	0.71	0.60	**0.95**	0.86	0.81
SMO	ZINC_random	**0.94**	**1.00**	**0.94**	**0.93**	**0.93**	0.87	**0.90**	**0.95**	0.87
	ZINC_diverse	**1.00**	**1.00**	**1.00**	**0.97**	0.78	0.74	**0.99**	0.86	0.86
	MDDR_random	**0.92**	**1.00**	**0.93**	**0.92**	**0.91**	0.85	0.77	0.83	0.63
	MDDR_diverse	**0.97**	**1.00**	**0.97**	**0.94**	0.84	0.78	**0.92**	0.80	0.72
	DUD_random	**1.00**	**1.00**	**1.00**	**0.95**	0.87	0.83	**1.00**	0.87	0.87
	DUD_diverse	**1.00**	**1.00**	**1.00**	**0.94**	0.87	0.82	**1.00**	0.88	0.88
Ibk	ZINC_random	**0.96**	**0.99**	**0.95**	**0.96**	0.79	0.73	**0.95**	0.83	0.79
	ZINC_diverse	**1.00**	**1.00**	**1.00**	**0.97**	0.71	0.65	**1.00**	0.69	0.65
	MDDR_random	**0.96**	**0.99**	**0.96**	**0.94**	0.78	0.72	**0.94**	0.70	0.61
	MDDR_diverse	**0.97**	**1.00**	**0.97**	**0.94**	0.77	0.70	**0.97**	0.64	0.54
	DUD_random	**1.00**	**1.00**	**1.00**	**0.96**	0.80	0.75	**0.98**	0.82	0.80
	DUD_diverse	**1.00**	**1.00**	**1.00**	**0.94**	0.78	0.71	**0.99**	0.82	0.81
Decorate	ZINC_random	0.87	**1.00**	0.88	0.88	0.87	0.77	0.87	0.86	0.75
	ZINC_diverse	**0.99**	**1.00**	**0.99**	**0.95**	0.65	0.54	**0.92**	0.80	0.72
	MDDR_random	**0.90**	**0.94**	0.86	0.88	0.82	0.71	0.84	0.82	0.68
	MDDR_diverse	**0.94**	**1.00**	**0.95**	**0.93**	0.71	0.62	**0.92**	0.80	0.72
	DUD_random	**1.00**	0.88	0.88	**0.90**	0.66	0.51	**0.95**	0.81	0.76
	DUD_diverse	**1.00**	**0.93**	**0.92**	**0.91**	0.71	0.60	**0.95**	0.86	0.81
Hyperpipes	ZINC_random	**1.00**	**0.99**	**0.99**	0.87	0.77	0.65	**0.99**	0.86	0.85
	ZINC_diverse	**1.00**	**1.00**	**1.00**	**0.91**	0.71	0.60	**0.99**	0.86	0.85
	MDDR_random	**1.00**	**1.00**	**1.00**	0.84	0.75	0.60	**0.99**	0.86	0.85
	MDDR_diverse	**1.00**	**1.00**	**1.00**	0.87	0.72	0.58	**0.99**	0.86	0.85
	DUD_random	**1.00**	**1.00**	**1.00**	**0.91**	0.66	0.52	**1.00**	0.85	0.85
	DUD_diverse	**1.00**	**1.00**	**1.00**	**0.90**	0.72	0.61	**1.00**	0.86	0.85
J48	ZINC_random	0.80	0.80	0.62	0.86	0.87	0.75	0.87	0.90	0.78
	ZINC_diverse	**0.99**	**0.99**	**0.98**	**0.92**	0.69	0.58	**0.99**	0.89	0.88
	MDDR_random	0.77	**0.96**	0.77	0.86	0.84	0.71	0.81	0.76	0.59
	MDDR_diverse	0.85	**0.99**	0.86	**0.90**	0.73	0.62	0.89	0.74	0.62
	DUD_random	**0.99**	**0.98**	**0.97**	0.88	0.84	0.73	**0.99**	0.86	0.85
	DUD_diverse	**1.00**	**0.96**	**0.95**	**0.94**	0.81	0.75	**1.00**	0.87	0.87
Random Forest	ZINC_random	0.88	**1.00**	0.90	**0.93**	**0.93**	0.86	**0.93**	**0.97**	**0.91**
	ZINC_diverse	**1.00**	**1.00**	**1.00**	**0.97**	0.75	0.70	**0.99**	**0.91**	**0.91**
	MDDR_random	0.86	**1.00**	0.88	**0.91**	**0.92**	0.84	0.88	**0.91**	0.81
	MDDR_diverse	**0.94**	**1.00**	**0.95**	**0.95**	0.82	0.78	**0.94**	0.90	0.85
	DUD_random	**1.00**	**1.00**	**1.00**	**0.94**	0.86	0.81	**0.99**	0.87	0.87
	DUD_diverse	**1.00**	**1.00**	**1.00**	**0.94**	0.87	0.82	**0.99**	0.88	0.88
